# Structural Characterization of a Novel Galactoarabinan from *Baphicacanthus cusia* and Its Protective Effects Against Oxidative Stress and Inflammation via the PI3K/Akt and Nrf2/HO-1 Signaling Axes

**DOI:** 10.3390/antiox15060770

**Published:** 2026-06-19

**Authors:** Zanwen Zuo, Chen Yang, Wenli Liang, Qian Zhang, Yuliang Wang, Xiao Sheng, Qizhang Li

**Affiliations:** 1 Innovative Drug R & D Center, Anhui Provincial Engineering Laboratory for Efficient Utilization of Featured Resource Plants, Huaibei Key Laboratory of Efficient Cultivation and Utilization of Resource Plants, College of Life Sciences, Huaibei Normal University, Huaibei 235000, China; zanwenzuo@foxmail.com (Z.Z.); ycycyc0223@163.com (C.Y.); 18056148967@163.com (W.L.); qianzhang-edu@foxmail.com (Q.Z.); 2 School of Agriculture and Biology, Plant Biotechnology Research Center, Fudan-SJTU-Nottingham Plant Biotechnology R&D Center, Shanghai Jiao Tong University, Shanghai 200240, China; wangyuliang@sjtu.edu.cn; 3 Department of Pharmaceutical Botany, School of Pharmacy, Naval Medical University, Shanghai 200433, China

**Keywords:** *Baphicacanthus cusia*, Nan-Ban-Lan-Gen, galactoarabinan, oxidative stress, immunomodulation, Zebrafish, PI3K/Akt signaling, Nrf2/HO-1 axis

## Abstract

The roots of *Baphicacanthus cusia* (Nees) Bremek, commonly known as Nan-Ban-Lan-Gen, have been used for a long time in traditional Chinese medicine to manage inflammatory and infectious diseases. However, the structural features and bioactive potential of its polysaccharides have not been extensively studied. In the present study, a novel homogeneous polysaccharide (BcP-b2) was isolated from the roots of *B. cusia*, and its bioactivity was evaluated using an activity-guided purification strategy. Multi-dimensional structural analysis identified BcP-b2 as a highly branched galactoarabinan with a molecular weight of ~38.1 kDa, featuring a well-defined backbone and a variety of side chains. In vitro and in vivo assays demonstrated that BcP-b2 attenuated the accumulation of reactive oxygen species (ROS) and enhanced the activities of endogenous antioxidant enzymes, including superoxide dismutase (SOD), catalase (CAT), and glutathione peroxidase (GSH-Px). Additionally, BcP-b2 activated macrophages under basal conditions and alleviated lipopolysaccharide (LPS)-induced cytotoxicity and inflammatory mediator release. Transcriptomic and Western blot analyses revealed that these dual effects were achieved through the simultaneous suppression of the PI3K/Akt inflammatory axis and activation of the Nrf2/HO-1 antioxidant pathway, concomitant with enhanced nuclear translocation of Nrf2. These findings provide a molecular basis for the ethno-pharmacological use of Nan-Ban-Lan-Gen and identify BcP-b2 as a promising candidate for further investigation as a potential therapeutic agent.

## 1. Introduction

Polysaccharides are a major class of bioactive macromolecules found widely in plants, fungi, and microorganisms. In addition to their structural functions, these complex carbohydrates have diverse pharmacological activities, including antioxidant, immunomodulatory, and prebiotic effects [1,2,3,4]. In Traditional Chinese Medicine (TCM), polysaccharides are increasingly recognized as essential components of therapeutic efficacy, serving not only as indicators of quality but also as key bioactive constituents that modulate host defense [5,6]. Herbs categorized as “heat-clearing and detoxifying” (Qing-Re-Jie-Du) in TCM often exhibit significant anti-inflammatory and antioxidant properties, which are partially attributed to their polysaccharide fractions [7,8].

*Baphicacanthus cusia* (Nees) Bremek, a perennial herb belonging to the Acanthaceae family, has been used in China for centuries, both as a source of natural indigo and as a medicinal plant. Its leaves, known as Da-Qing-Ye, and processed stems, called Qing-Dai, are documented in the *Pharmacopoeia* *of the People’s Republic of China* for their antipyretic and detoxifying indications. The dried roots, referred to as “Nan-Ban-Lan-Gen”, are specifically prescribed for inflammatory and infectious disorders [9,10]. Historically, investigations of *B. cusia* have focused on low-molecular-weight indole alkaloids, including indigo, indirubin, and tryptanthrin, which exhibit antileukemic and antiviral activities [11,12,13]. In contrast, the polysaccharide fraction of this species remains structurally and functionally undercharacterized.

To date, only one study has reported the isolation and characterization of two water-soluble polysaccharide fractions, BCP-1 and BCP-2, from the roots of *B. cusia* [14]. These fractions exhibit average molecular weights of 11.6 and 26.7 kDa and display in vitro antioxidant capacity as well as in vitro and in vivo anti-inflammatory activity. However, these investigations have primarily involved phenotypic screening based on cell-free radical-scavenging assays and crude cytoprotection tests. The structural analysis has been limited to techniques such as Fourier-transform infrared spectroscopy, gas chromatography, and monosaccharide composition analysis, leaving glycosidic linkage sequences, branching patterns, and three-dimensional conformations unresolved [14]. This lack of detailed structural understanding is significant, as the biological activities of polysaccharides are governed by their fine structural features, including molecular weight, monosaccharide sequence, and branching architecture [15]. Furthermore, the mechanisms underlying the antioxidant and anti-inflammatory effects of *B. cusia* polysaccharides are not explored at the signaling-pathway level.

In this study, we employed an activity-guided purification strategy to isolate a homogeneous polysaccharide fraction, designated BcP-b2, from the dried roots of *B. cusia*. Compared to the previously reported BCP fractions, BcP-b2 possessed a significantly higher molecular weight (~38.1 kDa) and a more complex monosaccharide composition. Through multi-dimensional structural analysis, including size exclusion chromatography (SEC) coupled with multi-angle laser light scattering (MALLS) and refractive index (RI) detection, methylation analysis coupled with gas chromatography–mass spectrometry (GC-MS), and two-dimensional nuclear magnetic resonance (NMR) spectroscopy, we determined that BcP-b2 was a highly branched galactoarabinan with a defined backbone and explicit side-chain sequences. To expand our understanding beyond the phenotypic focus of prior investigations, we integrated high-resolution structural elucidation with mechanistic investigation at the signaling-pathway level. Our findings reveal that BcP-b2 exhibited dual antioxidant and anti-inflammatory effects by concurrently suppressing the PI3K/Akt inflammatory axis and activating the Nrf2/HO-1 antioxidant pathway. We validated these protective actions in lipopolysaccharide (LPS)-challenged RAW 264.7 macrophages and in a menaquinone-induced oxidative stress zebrafish model. Collectively, this study provides the first high-resolution structural and mechanistic insight into a bioactive polysaccharide from *B. cusia* roots, establishing a molecular basis for the ethno-pharmacological use of Nan-Ban-Lan-Gen and identifying BcP-b2 as a promising candidate for further investigation as a potential therapeutic agent.

## 2. Materials and Methods

### 2.1. Materials and Reagents

“Nan-Ban-Lan-Gen” (the dried roots of *B. cusia*, designated BcR) was purchased from Bozhou Yuanshengtang Pharmaceutical Co., Ltd. (Bozhou, Anhui, China) and authenticated by internal transcribed spacer (ITS) sequencing. Analytical-grade reagents were purchased from Sinopharm Chemical Reagent Co., Ltd. (Shanghai, China) unless otherwise stated. Monosaccharide standards were obtained from Millipore Sigma (St. Louis, MO, USA). RAW 264.7 mouse macrophage cells were obtained from Beyotime Biotechnology (Shanghai, China). *Drosophila melanogaster w^1118^* flies (stock #5905) were obtained from the Bloomington Drosophila Stock Center (BDSC; Bloomington, IN, USA). Zebrafish (*Danio rerio*) were obtained from Hunter Biotech (Hangzhou, China). Antibodies against phosphorylated Akt (p-Akt), Akt, nuclear factor erythroid 2-related factor 2 (Nrf2), and heme oxygenase-1 (HO-1) were purchased from Proteintech (Wuhan, China). GAPDH and Lamin B1 antibodies were used as loading controls for cytoplasmic and nuclear fractions, respectively. A goat anti-mouse IgG secondary antibody was obtained from Sangon (Shanghai, China).

### 2.2. Molecular Identification of BcR

The taxonomic identity of BcR was authenticated by DNA barcoding of the nuclear ribosomal internal transcribed spacer (ITS) region, a core barcode widely used for medicinal plant identification [16]. The sample was pulverized in liquid nitrogen, and total genomic DNA was extracted using a Super Plant Genomic DNA Kit (Tiangen, Beijing, China) following the manufacturer’s instructions. The ITS region was amplified by PCR using the universal primers ITS_F (5′-TCCTCCGCTTATTGATATGC-3′) and ITS_R (5′-GGAAGTAAAAGTCGTAACAAGG-3′). The amplicons were sequenced, and the resulting consensus sequence was subjected to the online tool BLASTn analysis against the NCBI GenBank database. Multiple sequence alignment was performed using the ClustalW algorithm in MEGA11 (version 11.0). A neighbor-joining (NJ) phylogenetic tree was constructed, and branching robustness was evaluated by bootstrap analysis with 1000 replicates.

### 2.3. Isolation and Purification of Polysaccharides from BcR

The ground BcR sample was defatted with absolute ethanol overnight. Polysaccharides were extracted with hot water at a solid-to-liquid ratio of 1:10 (*w*/*v*) at 60 °C for 4 h. The aqueous extract was concentrated and precipitated with four volumes of anhydrous ethanol at 4 °C for 24 h. The precipitate was collected, redissolved in deionized water, and deproteinized using the Sevag method (chloroform:n-butanol, 4:1, *v*/*v*). The resulting aqueous phase was dialyzed against deionized water (MWCO: 3500 Da) and lyophilized to yield the crude polysaccharide (BcP). For further purification, BcP was dissolved in deionized water, loaded onto a DEAE Seplife FF anion-exchange column, and eluted stepwise with NaCl solutions (0, 0.1, 0.2, and 0.3 M). The target fraction was concentrated and further purified on a Sephacryl S-400 HR gel filtration column. The carbohydrate content was monitored by the phenol–sulfuric acid method [17].

### 2.4. Structural Analysis

#### 2.4.1. Molecular Weight (Mw) Evaluation

The absolute molecular weight and homogeneity of BcP-b2 were determined using high-performance size-exclusion chromatography coupled with multi-angle laser light scattering and refractive index detection (HPSEC-MALLS-RI). The analytical platform consisted of an UltiMate 3000 HPLC system (Thermo Fisher Scientific, Germering, Germany), a DAWN HELEOS-II MALS detector (Wyatt Technology, Santa Barbara, CA, USA), and an Optilab T-rEX RI detector (Wyatt Technology, Santa Barbara, CA, USA). Chromatographic separation was achieved using two Shodex OHpak columns (SB-805 HQ and SB-803 HQ, 300 × 8 mm; Showa Denko K.K.) connected in series and maintained at 45 °C. The mobile phase, composed of 0.1 M NaNO_3_ containing 0.02% NaN_3_ (*w*/*w*), was delivered at a constant flow rate of 0.6 mL/min. The polysaccharide sample was dissolved in the mobile phase to a final concentration of 1 mg/mL, filtered through a 0.45-μm membrane, and injected at a volume of 100 μL. The specific refractive index increment (*dn*/*dc*) of BcP-b2 in 0.1 M NaNO_3_ was 0.141 mL/g. Data acquisition and processing were performed using ASTRA 6.1 software (Wyatt Technology, Santa Barbara, CA, USA) to calculate the weight-average molecular weight (Mw), number-average molecular weight (Mn), and polydispersity index (Mw/Mn).

#### 2.4.2. Ultraviolet–Visible (UV-Vis) and Fourier-Transform Infrared (FT-IR) Spectroscopy

Ultraviolet–visible (UV-Vis) absorption spectra were recorded over a wavelength range of 200–1000 nm using a multifunctional microplate reader (Thermo Fisher Scientific, Waltham, MA, USA). The Fourier-transform infrared (FT-IR) spectrum of BcP-b2 was acquired using a Nicolet iZ-10 spectrometer (Thermo Fisher Scientific, Waltham, MA, USA) equipped with a DTGS KBr detector and a KBr beam splitter. The sample was prepared by grinding approximately 2 mg of BcP-b2 with 200 mg of spectroscopic-grade potassium bromide (KBr; Sigma, St. Louis, MO, USA) and pressing the mixture into a 1-mm-thick transparent pellet. Spectra were recorded over a frequency range of 4000–400 cm^−1^ at a resolution of 4.00 cm^−1^ with 32 co-added scans. Background correction was performed against a pure KBr pellet under identical conditions.

#### 2.4.3. Monosaccharide Composition Analysis

Prior to chromatographic analysis, approximately 5 mg of the BcP-b2 sample was completely hydrolyzed using 2 M trifluoroacetic acid (TFA) at 121 °C for 2 h. The resulting hydrolysate was evaporated to dryness under a nitrogen stream and co-evaporated twice with anhydrous methanol to remove residual TFA. The dried residue was subsequently reconstituted in deionized water and filtered through a 0.22-μm microporous membrane. The monosaccharide profile was determined using high-performance anion-exchange chromatography coupled with pulsed amperometric detection (HPAEC-PAD) on an ICS-5000+ system (Thermo Fisher Scientific, Waltham, MA, USA). Chromatographic separation was achieved using a Dionex CarboPac PA20 analytical column (150 × 3.0 mm, 10 μm) maintained at 30 °C. The mobile phase comprised ultrapure water (eluent A), 0.1 M NaOH (eluent B), and 0.1 M NaOH containing 0.2 M sodium acetate (eluent C), delivered at a constant flow rate of 0.5 mL/min. The gradient elution profile (A/B/C, *v*/*v*/*v*) was programmed as follows: 95:5:0 at 0 min, 85:5:10 at 26 min, 60:0:40 at 42.1 min, and 95:5:0 at 60 min. Identification and quantification of individual monosaccharides were performed by comparing the retention times and peak areas with those of external standard calibration curves [18].

#### 2.4.4. X-Ray Diffraction (XRD) Analysis

The crystallographic characteristics of BcP-b2 were evaluated using powder X-ray diffraction (PXRD) [19]. Prior to analysis, the polysaccharide sample was dried to a constant weight at 60 °C, pulverized, and sieved (100-mesh). Approximately 20 mg of the homogenized powder was uniformly packed into a glass sample holder to ensure a flat analytical surface. Diffractograms were acquired on an X’Pert Pro diffractometer (PANalytical, Almelo, The Netherlands) equipped with a Cu-Kα radiation source (λ = 0.15406 nm) operating at 40 kV and 40 mA, utilizing a NaI scintillation detector. The diffraction data were recorded over a 2θ range of 5° to 60° with a step size of 0.02° and a scanning rate of 4°/min. The optical instrumental configuration consisted of a 1-mm divergence slit, a 1-mm anti-scatter slit, and a 0.1-mm receiving slit.

#### 2.4.5. Scanning Electron Microscope (SEM) Analysis

The micro-morphological characteristics of BcP-b2 were evaluated using field-emission scanning electron microscopy (FE-SEM). Prior to imaging, the polysaccharide sample was sieved (100-mesh) and mounted onto an aluminum stub via double-sided conductive carbon tape. To prevent charging artifacts and enhance surface conductivity, the specimen was sputter-coated with a thin layer of gold. Imaging was subsequently performed on a Zeiss Merlin Compact FE-SEM (Carl Zeiss, Oberkochen, Germany) equipped with a Schottky thermal field-emission gun and an in-lens Duo secondary electron detector. The instrument was operated under high-vacuum conditions at an accelerating voltage of 1.0 kV.

#### 2.4.6. Methylation Analysis

The glycosidic linkage patterns of BcP-b2 were elucidated via methylation analysis, adapted from the method described by Needs and Selvendran [20]. Initially, 2–3 mg of the polysaccharide was dissolved in 500 μL of anhydrous dimethyl sulfoxide (DMSO). Following the addition of sodium hydroxide powder (1 mg), the suspension was incubated for 30 min to generate alkoxide ions. Subsequently, 50 μL of methyl iodide was introduced, and the methylation reaction proceeded for 1 h in the dark. The permethylated product was partitioned into 2 mL of dichloromethane following the addition of 1 mL of ultrapure water. The organic layer was washed completely with water to remove excess reagents and evaporated to dryness under a nitrogen stream. The permethylated derivative was subsequently hydrolyzed using 100 μL of 4 M TFA at 100 °C for 2 h. After removing TFA by evaporation at 30 °C, the hydrolysate was reduced by the consecutive addition of 50 μL of 2 M aqueous ammonia and 50 μL of 1 M sodium borodeuteride (NaBD_4_), with the reaction maintained at room temperature for 2.5 h. The reduction was quenched with 20 μL of glacial acetic acid. The resulting mixture was dried under nitrogen and co-evaporated twice with 250 μL of anhydrous methanol to remove residual borate. The partially methylated alditols were then acetylated using 250 μL of acetic anhydride at 100 °C for 2.5 h. The resulting partially methylated alditol acetates (PMAAs) were extracted with dichloromethane (500 μL), washed completely with water, and analyzed via gas chromatography–mass spectrometry (GC-MS) using an Agilent 6890A-5977B system (Agilent Technologies, Santa Clara, CA, USA). Chromatographic separation was achieved on a TG-200 capillary column (30 m × 0.25 mm, 0.25 μm film thickness; SGE, Ringwood, Australia). Helium was employed as the carrier gas at a constant flow rate of 1.5 mL/min with a split ratio of 10:1. A 1 μL aliquot was injected. The column oven temperature program was initialized at 150 °C (held for 1 min), ramped to 210 °C at 2 °C/min (held for 2 min), and finally elevated to 240 °C at 2 °C/min (held for 3 min). Mass spectra were acquired in full-scan mode (*m*/*z* 50–350) utilizing an electron ionization (EI) energy of 50 eV. The PMAAs were identified by matching their retention times and characteristic mass fragmentation patterns with standard library data, and their relative molar ratios were calculated based on peak areas normalized to their respective effective carbon responses.

#### 2.4.7. Nuclear Magnetic Resonance (NMR) Analysis

A dried sample BcP-b2 was dissolved in deuterium oxide (D_2_O). A Bruker AVANCE NEO 500M spectrometer system (Bruker, Rheinstetten, Germany) was used to record 1D- and 2D-NMR (^1^H-NMR, ^13^C-NMR, COSY, NOESY, HMBC, and HSQC) spectra.

### 2.5. Assessment of Antioxidant Ability In Vitro

The radical scavenging capacities of 1,1-diphenyl-2-picrylhydrazyl (DPPH), hydroxyl radical (•OH), and superoxide anion (•O_2_^−^) were evaluated in vitro using the following assay kits: the DPPH Free Radical Scavenging Capacity Assay Kit (Sangon, Shanghai, China), Hydroxyl Free Radical Scavenging Capacity Assay Kit (Sangon, Shanghai, China), and Superoxide Anion Radical Scavenging Capacity Assay Kit (Mlbio, Shanghai, China), respectively. The absorbance data were acquired using a microplate reader (Supermax 3100; Flash Biotech, Shanghai, China).

### 2.6. Animal Experiments

#### 2.6.1. Drosophila Survival Assay

The *w^1118^* flies were maintained in an incubator at 25 °C with 65% relative humidity under a 12-h light–dark cycle on standard agarose–cornmeal medium. For the survival assay, five experimental groups (*n* = 120 per group) were established: control, model (4% DSS), low-dose (L; 0.5 mg/mL BcP), medium-dose (M; 1 mg/mL BcP), and high-dose (H; 2 mg/mL BcP). Age-synchronized female flies (approximately 3 days post-eclosion) were sex-segregated and reared on a regular diet, and then starved for 2 h to promote ingestion prior to treatment. The flies were subsequently transferred into experimental vials. The control group was administered 5% (*w*/*v*) sucrose. The model group was administered 5% (*w*/*v*) sucrose supplemented with 4% (*w*/*v*) DSS to induce intestinal damage [21]. The experimental groups were maintained on regular medium containing BcP at the indicated concentrations for 7 days, followed by administration of 5% (*w*/*v*) sucrose supplemented with 4% (*w*/*v*) DSS. Survival was monitored daily, and fresh medium was provided every 2–3 days. Three independent biological replicates were performed.

#### 2.6.2. Zebrafish Experiments

All in vivo zebrafish assays were conducted at Hunter Biotech (Hangzhou, China; AAALAC International certification no.: 001458; experimental animal production license no.: SYXK (Zhe) 2022-0004) in strict accordance with the ARRIVE guidelines and under the ethical approval of the Institutional Animal Care and Use Committee (approval no.: IACUC-2024-10205-01). Zebrafish embryos were maintained in standard embryo medium at 28 °C under a 14-h light/10-h dark cycle. To determine the maximum tolerated concentration (MTC), wild-type AB strain larvae at 3 days post-fertilization (dpf) were seeded into six-well plates (30 larvae/well in 3 mL of medium). The larvae were pretreated with BcP-b2 (125, 250, 500, 1000, or 2000 μg/mL) for 3 h, followed by a 24-h exposure to menaquinone to induce oxidative stress (excluding the vehicle control). Mortality and morphological anomalies were monitored at 12-h intervals, strictly adhering to OECD Test Guideline 236 (Fish Embryo Acute Toxicity Test). Assessed parameters included apical lethal endpoints (coagulation, absence of somite formation, non-detachment of the tail, and lack of heartbeat) and sublethal developmental toxicities (pericardial edema, yolk sac edema, and spinal curvature). No mortality or teratogenic phenotypes were observed at any tested concentration (0% lethality; *n* = 30 per group), thereby establishing 2000 μg/mL as the maximum non-lethal concentration. Consequently, BcP-b2 concentrations of 500, 1000, and 2000 μg/mL were selected to evaluate in vivo antioxidant efficacy. Melanin-deficient mutant (*albino*) larvae at 3 dpf were assigned to experimental cohorts via a computer-generated randomization sequence: vehicle control, menaquinone-induced model, positive control (62.5 μg/mL *N*-acetylcysteine, NAC), and BcP-b2 treatment groups. Following the established 3-h pretreatment and subsequent 24-h menaquinone challenge, intracellular reactive oxygen species (ROS) accumulation was quantified using the CellROX Green fluorescent probe (Invitrogen, Carlsbad, CA, USA). Post-staining, the larvae were washed thoroughly to remove extracellular dye. Ten randomly selected larvae per cohort were anesthetized using 0.16% tricaine methanesulfonate and visualized under an AZ100 fluorescence microscope (Nikon, Tokyo, Japan). To preclude observation bias, investigators were blinded to group allocations during both image acquisition and subsequent quantitative analysis. The integrated fluorescence intensity within the yolk sac region was calculated using NIS-Elements D 3.20 software (Nikon, Tokyo, Japan).

### 2.7. Cell Experiments

#### 2.7.1. Cell Culture

The murine macrophage cell line RAW 264.7 was cultured in high-glucose Dulbecco‘s Modified Eagle Medium (DMEM) supplemented with 10% (*v*/*v*) fetal bovine serum (FBS) and 1% (*v*/*v*) penicillin–streptomycin solution (100 U/mL penicillin and 100 μg/mL streptomycin) [22]. The cells were maintained at 37 °C in a humidified incubator with a 5% CO_2_ atmosphere. The culture medium was replenished every 2–3 days. Upon reaching approximately 80% confluence, the cells were detached using 0.25% trypsin–EDTA and subcultured at a split ratio of 1:3 to 1:5. Only cells in the exponential growth phase and within 20 passages were utilized for all downstream experiments.

#### 2.7.2. Cell Viability Study Assay

Cell viability was evaluated using a Cell Counting Kit-8 (CCK-8) assay (Yeasen, Shanghai, China). RAW 264.7 macrophages in the exponential growth phase were seeded into 96-well plates at a density of 2 × 10^4^ cells/well in 100 μL of complete medium. The plates were incubated at 37 °C in a humidified atmosphere with 5% for 12–24 h to facilitate cell attachment. Subsequently, the culture medium was aspirated and replaced with fresh medium containing designated concentrations of BcP-b2, followed by an additional 24-h incubation. To assess cytoprotection against LPS-induced injury, the cells were pretreated with BcP-b2 for 24 h, followed by a 24-h challenge with 1 μg/mL LPS. Following the respective treatments, a 10 μL aliquot of CCK-8 reagent was added to each well, and the plates were incubated in the dark at 37 °C for 2 h. The optical density (OD) was measured at 450 nm using a microplate reader. Each biological replicate was performed with six technical replicates.

#### 2.7.3. Determination of Antioxidant Enzymes in RAW 264.7 Cells

After pretreatment with BcP-b2 for 24 h, the cells were exposed to LPS for an additional 24 h. The activities of superoxide dismutase (SOD), catalase (CAT), and glutathione peroxidase (GSH-Px) were determined using the Total Superoxide Dismutase Assay Kit with WST-8, the Catalase Assay Kit, and the Cellular Glutathione Peroxidase Assay Kit with NADPH (Beyotime, Shanghai, China) following the manufacturer’s instructions, respectively. Data represent three independent biological replicates.

#### 2.7.4. Detection of Accumulated ROS in RAW 264.7 Cells

Intracellular ROS levels were measured using a Reactive Oxygen Species Assay Kit (Beyotime, Shanghai, China) based on the DCFH-DA fluorescent probe. After pre-treatment with BcP-b2 for 24 h, the cells were challenged with LPS (1 μg/mL) for an additional 24 h. The culture medium was removed, and the cells were incubated with DCFH-DA (10 μM, diluted 1:1000 in serum-free medium) at 37 °C for 20 min in the dark. The cells were then washed three times with PBS to remove extracellular probes. Fluorescence images were captured using a fluorescence microscope. Experiments were conducted with three independent biological replicates.

#### 2.7.5. Relative Expressions of Inflammatory Mediators

The levels of inflammatory and antioxidant mediators in cells treated with BcP-b2 were detected using quantitative real-time polymerase chain reaction (qRT-PCR). cDNA was prepared using FreeZol Reagent (Vazyme, Nanjing, Jiangsu, China). qRT-PCR was performed using the TB Green^®^ Premix Ex Taq™ II (TaKaRa, Beijing, China). The primers used are listed in Table S1. Three independent biological replicates were analyzed for each group (*n* = 3).

#### 2.7.6. RNA Sequencing

Total RNA was isolated from RAW 264.7 macrophages using the TaKaRa MiniBEST Universal RNA Extraction Kit (TaKaRa Bio, Beijing, China) in accordance with the manufacturer’s protocol. RNA integrity was evaluated utilizing a 5300 Bioanalyzer (Agilent Technologies, Santa Clara, CA, USA), and only samples exhibiting an RNA integrity number (RIN) greater than 6.5 were advanced to library construction. Transcriptome libraries were prepared utilizing the Illumina Stranded mRNA Prep Ligation Kit (Illumina, San Diego, CA, USA). Briefly, mRNA was enriched from 1 μg of total RNA using poly-T oligo-attached magnetic beads, chemically fragmented, and reverse-transcribed into first-strand cDNA. Second-strand cDNA synthesis incorporated dUTP to preserve strand specificity. Following end-repair, 3′-adenylation, and adapter ligation, the libraries underwent PCR amplification and size selection to yield cDNA fragments of approximately 300 bp. Library concentration was quantified with a Qubit 4.0 Fluorometer (Thermo Fisher Scientific, Waltham, MA, USA), and sequencing was executed on a NovaSeq X Plus platform (Illumina) in a paired-end 150-bp (PE150) configuration, yielding approximately 50 million raw reads per sample. Raw sequencing data were subjected to quality control and adapter trimming using fastp (v0.23.4) under default parameters. The resulting clean reads were mapped to the mouse reference genome using HISAT2 (v2.1.0). Transcript assembly was performed with StringTie (v2.1.7) employing a reference-based strategy. Gene expression quantification was performed with RSEM (v1.3.3), normalizing transcript abundance to transcripts per million (TPM). Differential expression analysis was executed using the DESeq2 package (v1.44.0), which applies a median-of-ratios method for size factor normalization. Differentially expressed genes (DEGs) were rigorously defined applying statistical thresholds of a false discovery rate (FDR)-adjusted *p*-value (FDR) < 0.05 and an absolute Log_2_ fold change (Log_2_FC) > 2. Each experimental cohort comprised three independent biological replicates.

#### 2.7.7. PPI Network Construction

From the 1,353 differentially expressed genes (DEGs) identified, a focused subset of 20 key genes (*IL-1β*, *IL6*, *CCL2*, *CCL7*, *CXCL2*, *CXCL3*, *PTGS2*, *IL10*, *IL33*, *CSF2*, *LCN2*, *MMP13*, *GSTP3*, *NFE2L2*, *HMOX1*, *PIK3IP1*, *CX3CR1*, *IL21R*, *PDCD4*, and *ABCA1*) was prioritized through a systematic four-tier filtering strategy to ensure objectivity and reproducibility. First, we extracted all DEGs that were annotated to Gene Ontology (GO) biological-process terms significantly enriched (FDR-adjusted *p* < 0.05) for inflammatory response, immune response, oxidative stress response, or reactive oxygen species metabolic process (Table S4), as well as genes mapped to significantly enriched Kyoto Encyclopedia of Genes and Genomes (KEGG) pathways, specifically Toll-like receptor signaling, PI3K-Akt signaling, the TNF signaling pathway, and the IL-17 signaling pathway (Table S5). Second, within the DEG pool already defined by the criteria in Section 2.7.6 (|Log_2_FC| > 2 and FDR-adjusted *p* < 0.05), we prioritized the most robustly regulated transcripts by ranking fold-change magnitudes, retaining the top-ranking candidates with the strongest transcriptional responses for subsequent pathway-relevance curation. Third, we performed a literature-based manual curation to retain only those genes with direct experimental evidence for functional roles in macrophage immunomodulation and redox homeostasis. The retained genes were classified into four functional categories: (i) pro-inflammatory cytokine and chemokine signaling (*IL-1β*, *IL6*, *CCL2*, *CCL7*, *CXCL2*, *CXCL3*, *PTGS2*, *CSF2*, *LCN2*, *MMP13*); (ii) anti-inflammatory or resolution-phase mediators (*IL10*, *IL33*); (iii) Nrf2-driven antioxidant defense (*NFE2L2*, *HMOX1*, *GSTP3*); and (iv) negative regulators of the PI3K/Akt axis and related signaling nodes (*PIK3IP1*, *PDCD4*, *CX3CR1*, *IL21R*, *ABCA1*). This pathway-relevance filter yielded the final set of 20 genes. Fourth, the network size was capped at 20 nodes to balance statistical power with visual interpretability, a convention consistent with previous macrophage transcriptomic network analyses. To explore the interactions among these prioritized genes, we queried them against the Search Tool for the Retrieval of Interacting Genes/Proteins (STRING) database (v12.0) to construct a protein–protein interaction (PPI) network [23]. Network edges representing medium-confidence interactions (minimum required interaction score > 0.4) were retained. The integrated network was then exported to Cytoscape software (v3.9.1) for visualization and topological analysis [24]. Hub genes were ranked using the Maximal Clique Centrality (MCC) algorithm from the cytoHubba plugin [25].

#### 2.7.8. Western Blotting (WB)

As previously reported [26], cells were treated with BcP-b2, and total proteins were extracted using RIPA lysis buffer (Beyotime, Shanghai, China) supplemented with a protease and phosphatase inhibitor cocktail (Beyotime, Shanghai, China). Nuclear and cytoplasmic proteins were extracted with the Nuclear and Cytoplasmic Protein Extraction Kit (Beyotime, Shanghai, China). Protein quantification was carried out using the BCA Protein Assay Kit (Sangon, Shanghai, China). The protein bands on the membrane were visualized using BeyoECL SuperMoon (Beyotime, Shanghai, China). GAPDH was used as an internal reference, and quantitative analyses were conducted using ImageJ (v1.54d).

### 2.8. Statistics Analysis

All experiments were conducted in at least three independent biological replicates, and quantitative data are expressed as the mean ± standard deviation (SD). Biological replicates are defined as independent experiments performed on different batches of cells, animals, or plant samples. Technical replicates are defined as repeated measurements of the same biological sample. Technical replicates were performed for each biological replicate. The resulting biological replicate means were used for statistical analysis and graphical presentation. Statistical evaluations were performed using GraphPad Prism software (version 10.6.0; GraphPad Software, San Diego, CA, USA). The normality of data distributions and the homogeneity of variances were assessed using the Shapiro–Wilk test and Levene’s test, respectively. Datasets that met parametric assumptions were analyzed with a two-tailed, unpaired Student’s *t*-test for pairwise comparisons, or a one-way analysis of variance (ANOVA) followed by Tukey’s *post hoc* test for comparisons among multiple groups. For non-parametric datasets, the Mann–Whitney U test was used for two-group comparisons, while the Kruskal–Wallis test followed by Dunn’s *post hoc* comparison was used for multiple groups. A probability (*p*) value of <0.05 was considered statistically significant. Significance levels in figures are denoted as follows: *, *p* < 0.05; **, *p* < 0.01; ***, *p* < 0.001; and ****, *p* < 0.0001.

## 3. Results

### 3.1. Molecular Authentication of BcR

**Figure 1:**
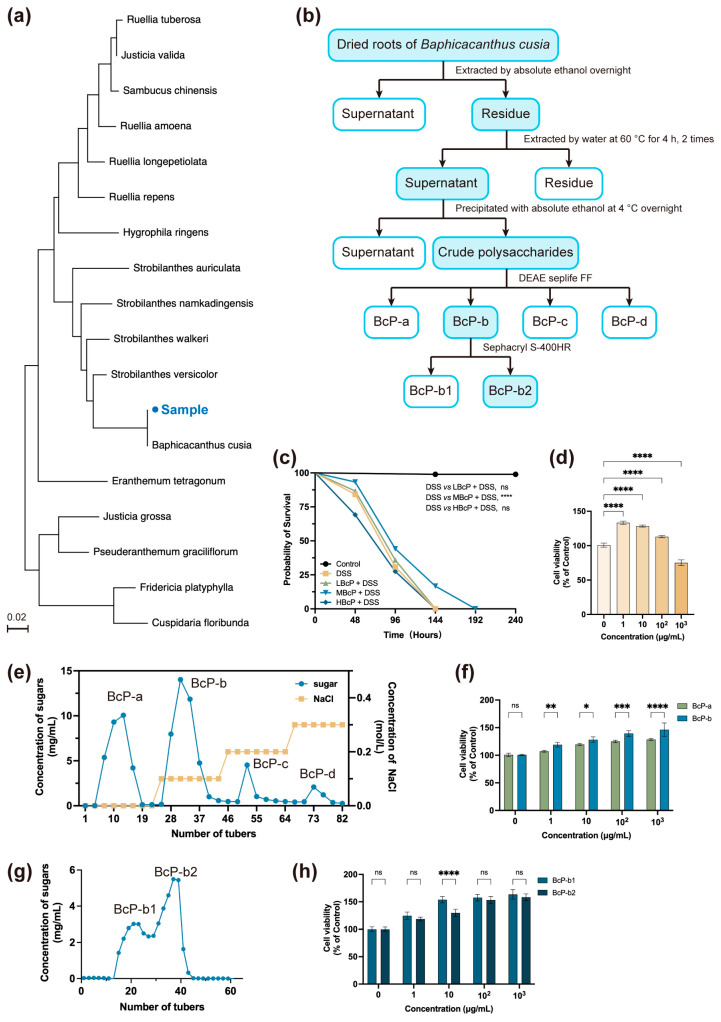
Isolation and purification of BcP-b2. (**a**) Molecular phylogenetic analysis of the dried *Baphicacanthus cusia* roots (BcR) and its closely related taxa. A multiple sequence alignment of the internal transcribed spacer (ITS) region was performed using ClustalW, and a phylogenetic tree was generated using the neighbor-joining (NJ) method with 1000 bootstrap replicates utilizing MEGA 11 software. (**b**) Schematic representation of the sequential extraction and fractionation protocol for BcP-b2. (**c**) Survival curves of female *Drosophila melanogaster* (*w^1118^* strain) subjected to dextran sulfate sodium (DSS)-induced stress. Flies were maintained on either a vehicle control diet or basal diets supplemented with BcP at low (0.5 mg/mL, LBcP), medium (1 mg/mL, MBcP), or high (2 mg/mL, HBcP) concentrations. The assay comprised three independent biological replicates, with a total of 120 flies per group. The assay was independently replicated three times. Statistical significance was evaluated using the Gehan–Breslow–Wilcoxon test. ns, no significant difference (*p* > 0.05); ****, *p* < 0.0001. (**d**) Effect of the crude extract BcP on the cellular viability of RAW 264.7 macrophages. (**e**) Anion-exchange chromatographic elution profile of BcP on a DEAE-Seplife FF column. (**f**) Cellular viability assessment of intermediate fractions BcP-a and BcP-b in RAW 264.7 macrophages. (**g**) High-resolution size-exclusion chromatogram of fraction BcP-b on a Sephacryl S-400 HR column, yielding subfractions BcP-b1 and BcP-b2. (**h**) Cellular viability assessment of purified subfractions BcP-b1 and BcP-b2 in RAW 264.7 macrophages. For panels (**d**,**f**,**h**), cells were treated with the indicated polysaccharide concentrations for 24 h in DMEM with 10% FBS at 37 °C and 5% CO_2_. Data are expressed as the mean ± standard deviation (SD) derived from six technical replicates per condition, pooled from three independent biological experiments. Statistical significance was assessed using a one-way analysis of variance (ANOVA) followed by Tukey’s *post hoc* multiple comparisons test using GraphPad Prism 10. ns, no significant difference (*p* > 0.05); *, *p* < 0.05; **, *p* < 0.01; ***, *p* < 0.001; and ****, *p* < 0.0001.

Accurate taxonomic identification of the botanical starting material is essential for conducting reliable phytochemical investigations. Herein, the internal transcribed spacer (ITS) region, a universally recognized core DNA barcode for medicinal plants, was utilized to verify the botanical origin of the dried *Baphicacanthus cusia* roots [16]. After PCR amplification and sequencing, the resulting sequence exhibited 100% pairwise identity with the reference ITS sequence of *B. cusia* via BLASTn analysis against the NCBI GenBank database (JX443747.1). Further phylogenetic analysis using the neighbor-joining (NJ) method demonstrated that the BcR sample clustered into a monophyletic clade with *B. cusia* (Figure 1a). It was clearly distinguished from other closely related species. Collectively, these molecular phylogenetic data definitively verified the plant material, establishing a validated botanical origin for the subsequent isolation and structural elucidation of the polysaccharide BcP-b2.

### 3.2. Isolation and Purification of BcP-b2

The workflow for sequential extraction and purification is illustrated in Figure 1b. The initial extraction of dried roots (BcR; 1.2 kg) yielded 77.3 g of crude polysaccharide (BcP), resulting in a mass fraction yield of 6.44% (*w*/*w*). To isolate the specific macromolecular constituents responsible for the ethnopharmacological efficacy of *B. cusia*, BcP was subjected to an activity-guided fractionation strategy. In vivo assessment using a dextran sulfate sodium (DSS)-induced *Drosophila* intestinal injury model revealed that 1 mg/mL BcP significantly attenuated inflammatory lesions and extended lifespan (*p* < 0.0001; Figure 1c). Concurrently, in vitro evaluation in RAW 264.7 macrophages indicated that BcP maintained cellular viability at exposure concentrations ranging from 0 to 100 μg/mL, whereas pronounced cytotoxicity emerged at 1000 μg/mL (Figure 1d).

Based on these phenotypic observations, the crude extract was fractionated using anion-exchange chromatography with a DEAE-Seplife FF column, resulting in four distinct fractions (BcP-a to BcP-d). Fraction BcP-b was prioritized for further purification due to its highest total carbohydrate content (91.6%) and minimal cytotoxicity towards RAW 264.7 macrophages (Figure 1e,f). Subsequent size-exclusion chromatography of BcP-b on a Sephacryl S-400 HR column yielded two homogeneous subfractions, designated BcP-b1 and BcP-b2 (Figure 1g). Although both subfractions exhibited comparable biocompatibility profiles (Figure 1h), BcP-b2 demonstrated superior free-radical scavenging efficacy in preliminary cell-free antioxidant assays (Table S2). Consequently, BcP-b2 was selected as the optimal candidate for comprehensive structural elucidation and mechanistic interrogation.

### 3.3. Chemical and Structural Characterization

#### 3.3.1. UV Spectrum Analysis

**Figure 2:**
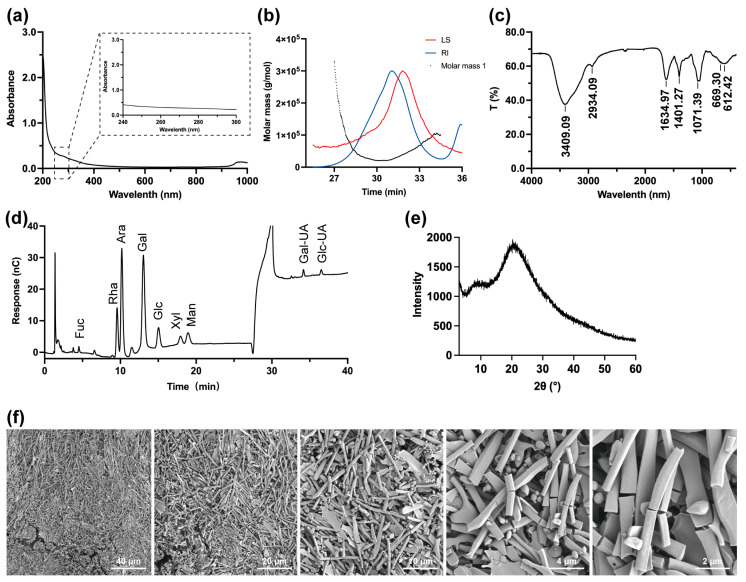
Chemical and morphological features of BcP-b2. (**a**) Ultraviolet–visible (UV-Vis) absorption spectrum over the wavelength range of 200–1000 nm. (**b**) Absolute molecular weight (Mw) determination using HPSEC-MALLS-RI. The specific refractive index increment (dn/dc) was set at 0.141 mL/g. Red line, multi-angle laser light scattering (MALS) signal; blue line, differential refractive index (dRI) signal; black dots, calculated molar mass distribution. (**c**) Fourier-transform infrared (FT-IR) spectrum spanning the frequency range of 4000–400 cm^−1^. (**d**) High-performance anion-exchange chromatography with pulsed amperometric detection (HPAEC-PAD) chromatogram for monosaccharide composition analysis. Ara, arabinose; Fuc, fucose; Gal, galactose; Gal-UA, galacturonic acid; Glc, glucose; Glc-UA, glucuronic acid; Man, mannose; Rha, rhamnose; Xyl, xylose. Quantitative data are presented as the mean ± standard deviation (SD) derived from three independent acid hydrolysis replicates. (**e**) Powder X-ray diffraction diffractogram recorded over a 2θ range of 5° to 60°. (**f**) Field-emission scanning electron microscopy (FE-SEM) micrographs detailing the microscopic topography. Scale bars are indicated in the respective panels.

The ultraviolet–visible (UV-Vis) absorption spectrum of BcP-b2 was recorded over a wavelength range of 200–1000 nm (Figure 2a). The spectrum did not show any observable absorption peaks at 260 and 280 nm, which are typically associated with nucleic acids and proteins, respectively. This lack of absorption maxima confirms the effective removal of these macromolecular contaminants during the sequential extraction and purification protocols. As a result, it establishes the requisite high purity of BcP-b2 for downstream structural elucidation.

#### 3.3.2. Mw Analysis

The molar mass distribution of BcP-b2 was determined using HPSEC-MALLS-RI. The resulting refractive index chromatogram exhibited a single, highly symmetric elution peak (Figure 2b), suggesting a narrow molecular weight distribution. The absolute weight-average molecular weight (Mw) and number-average molecular weight (Mn) were calculated as ~38.1 kDa and ~30.4 kDa, respectively. As a result, the polydispersity index (Mw/Mn) was determined to be 1.253. This relatively low index indicates that BcP-b2 consists of a highly homogeneous polysaccharide population without significant intermolecular aggregation or degradation.

#### 3.3.3. FT-IR Spectrum Analysis

The FT-IR spectrum, as illustrated in Figure 2c, reveals a typical absorption profile characteristic of acidic polysaccharides. A broad and intense absorption band centered at 3409 cm^−1^ is associated with the O-H stretching vibrations from inter- and intramolecular hydrogen bonds within the polysaccharide matrix. A minor signal at 2934 cm^−1^ corresponds to the C-H stretching vibrations of the carbohydrate backbone. Two prominent absorption peaks observed at 1635 cm^−1^ and 1401 cm^−1^ are attributed to the asymmetric and symmetric stretching vibrations of carboxylate groups (COO^−^), respectively, confirming the presence of uronic acids, such as galacturonic and glucuronic acids, identified in the monosaccharide composition analysis [27,28]. It is noteworthy that the 1635 cm^−1^ band overlaps with the characteristic Amide I region of proteins. However, the complete absence of a corresponding Amide II band at ~1540 cm^−1^ confirms the effective depletion of proteinaceous contaminants, verifying the high purity of BcP-b2. Additionally, a strong absorption band at 1071 cm^−1^ within the carbohydrate “fingerprint” region (1200–1000 cm^−1^) is diagnostic of the coupled C-O-C and C-O-H stretching vibrations in pyranose rings and glycosidic linkages [29,30]. Furthermore, minor absorption bands at 669 cm^−1^ and 612 cm^−1^ are assigned to the out-of-plane bending vibrations of O-H or the skeletal modes of the pyranose rings.

#### 3.3.4. Monosaccharide Composition Analysis

Chromatographic profiling using HPAEC-PAD (Figure 2d) revealed that BcP-b2 is an acidic heteropolysaccharide. The monosaccharide composition consisted of galactose (Gal, 33.12%), arabinose (Ara, 29.17%), rhamnose (Rha, 16.68%), glucose (Glc, 6.65%), mannose (Man, 4.52%), xylose (Xyl, 3.78%), galacturonic acid (GalA, 3.33%), glucuronic acid (GlcA, 1.65%), and fucose (Fuc, 1.09%). Gal and Ara were the predominant monosaccharides, collectively accounting for 62.29% of the total molar ratio. This composition establishes the primary structural framework of BcP-b2 as a galactoarabinan-type polysaccharide. Furthermore, the presence of minor neutral sugars (Rha, Xyl, and Man) alongside uronic acids suggests a complex structure with various side-chain substitutions, a characteristic that was further confirmed by methylation analysis.

#### 3.3.5. X-Ray Analysis

The crystallographic properties of BcP-b2 were evaluated using powder X-ray diffraction. As depicted in Figure 2e, the resulting diffractogram exhibited a broad dispersion halo centered at 2θ = 20.24°, accompanied by a minor, weak diffraction signal at 2θ = 8.98°. The lack of sharp, intense Bragg reflections indicates that BcP-b2 primarily exists as an amorphous macroscopic matrix, with localized semi-crystalline domains.

#### 3.3.6. SEM Analysis

The microscopic topography of BcP-b2 was characterized using field-emission scanning electron microscopy (FE-SEM). The acquired micrographs (Figure 2f) revealed a heterogeneous morphological profile, comprising well-defined rod-like structures interspersed with irregular spherical aggregates. Furthermore, these solid matrices exhibited predominantly smooth surface textures without visible porosity, which is characteristic of the compact, non-covalent intermolecular aggregation commonly observed in lyophilized high-molecular-weight polysaccharides.

#### 3.3.7. Linkage and NMR Analyses

The glycosidic linkage patterns of BcP-b2 were systematically elucidated via methylation analysis, followed by GC-MS analysis of the resulting partially methylated alditol acetates (PMAAs). A total of 24 distinct linkage types were identified and quantified based on their characteristic retention times and relative molar ratios (Table 1). The predominant linkages included →3,6)-β-D-Gal*p*-(1→ (14.66%), terminal α-L-Ara*f* (10.40%), terminal β-D-Gal*p* (9.23%), →6)-β-D-Gal*p*-(1→ (7.22%), →2)-α-L-Rha*p*-(1→ (5.94%), →5)-α-L-Ara*f*-(1→ (5.56%), α-L-Rha*p* (5.41%), →3)-β-D-Gal*p*-(1→ (4.91%), →2,4)-α-L-Rha*p*-(1→ (4.69%), →3-β-D-Man*p*-(1→ (4.11%), terminal β-D-Glc*p* (4.64%), and →4)-β-D-Xyl*p*-(1→ (2.92%). Independent validation was achieved through electron-impact mass spectrometry (EI-MS) fragmentation patterns of the PMAAs (Supplementary Data). The substantial presence of diverse branching residues, along with a similar abundance of terminal sugar residues, indicates a highly branched molecular architecture typical of a complex galactoarabinan.

**Figure 3:**
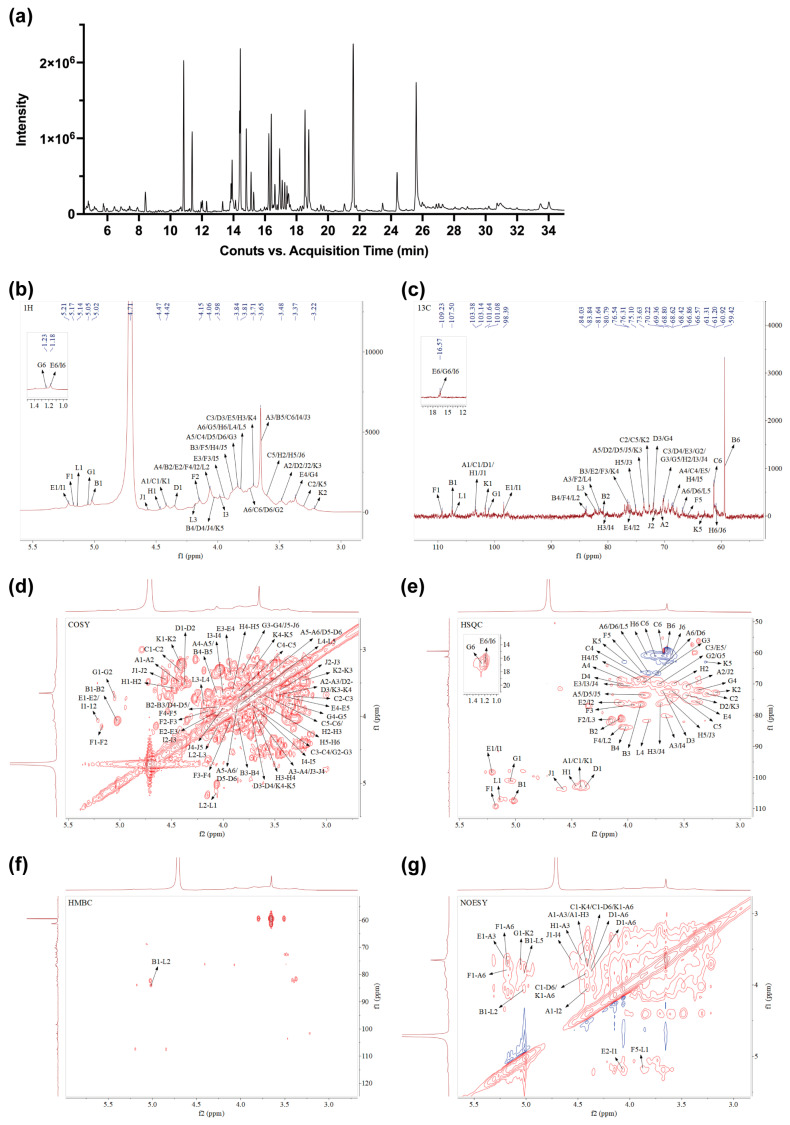
Structural characterization of BcP-b2 using multi-dimensional NMR and GC-MS analysis. (**a**) Total ion chromatogram (TIC) of partially methylated alditol acetates (PMAAs) obtained from the methylation analysis of BcP-b2. This was acquired using an Agilent 6890A-5977B GC-MS system equipped with a TG-200 capillary column (30 m × 0.25 mm × 0.25 μm). (**b**–**g**) Nuclear magnetic resonance (NMR) spectra recorded in D_2_O at 25 °C on a Bruker AVANCE NEO 500 MHz spectrometer: (**b**) ^1^H NMR spectrum; (**c**) ^13^C NMR spectrum; (**d**) COSY spectrum; (**e**) HSQC spectrum; (**f**) HMBC spectrum; and (**g**) NOESY spectrum.

To determine the precise sequences, anomeric configurations, and substitution patterns of individual monosaccharide units, we conducted comprehensive 1D and 2D NMR spectroscopy using a Bruker AVANCE NEO 500 MHz spectrometer at 25 °C in D_2_O. The ^1^H NMR spectrum displayed a range of anomeric signals between δ 4.30–5.40 ppm, with corresponding anomeric carbon resonances ranging from δ 98.0 to 110.0 ppm in the ^13^C NMR spectrum, indicating the simultaneous presence of both α- and β-anomeric configurations. We unambiguously identified twelve distinct anomeric spin systems, designated as residues **A** through **L**, based on HSQC cross-peaks at 4.42/103.38 (**A**), 5.02/107.50 (**B**), 4.41/103.24 (**C**), 4.37/103.44 (**D**), 5.21/98.39 (**E**), 5.17/109.23 (**F**), 5.05/101.08 (**G**), 4.47/103.14 (**H**), 5.22/98.70 (**I**), 4.57/103.78 (**J**), 4.40/101.64 (**K**), and 5.14/106.98 (**L**) ppm (Figure 3b,c; Table 2).

The intra-residue spin systems were thoroughly mapped by tracing scalar coupling connectivities in the COSY spectrum (Figure 3d) from the anomeric proton through to H6. For residue **A**, consecutive cross-peaks at δ 4.42/3.48 (H1/H2), 3.48/3.66 (H2/H3), 3.66/4.07 (H3/H4), 4.07/3.86 (H4/H5), and 3.86/3.74, 3.82 (H5/H6) permitted a complete proton assignment. The HSQC spectrum (Figure 3e) subsequently correlated these protons with their respective carbons at δ 103.38 (C1), 70.73 (C2), 80.18 (C3), 68.41 (C4), 73.64 (C5), and 66.57 (C6). The pronounced downfield shifts of C3 (δ 80.18) and C6 (δ 66.57) relative to unsubstituted galactose dynamically confirmed substitution at the O-3 and O-6 positions. Concurrently, the anomeric carbon chemical shift (δ > 100 ppm) and the characteristic ^3^*J*_H1,H2_ coupling pattern corroborated a β-configuration. Consequently, residue **A** was definitively assigned as →3,6)-β-D-Gal*p*-(1→. Residues **B** through **L** were systematically characterized using a similar analytical approach (Table 2).

The architectural sequence and inter-residue connectivities were established using HMBC and NOESY spectra, exploiting both through-bond scalar couplings and through-space dipolar relaxations. A crucial trans-glycosidic HMBC cross-peak at δ 5.02/83.93 (**B** H1/**L** C2) verified the linkage between the terminal α-L-Ara*f* (**B**) and →2,5)-α-L-Ara*f* (**L**). Additionally, intense NOESY cross-peaks provided essential evidence of spatial proximity for the backbone and branch-point structures. Specifically, inter-residue spatial correlations from **E** H1 to **A** H3 (δ 5.21/3.66) and **H** H1 to **A** H3 (δ 4.47/3.66) indicated that →2)-α-L-Rha*p* (**E**) and →3)-β-D-Gal*p* (**H**) were connected to the O-3 position of →3,6)-β-D-Gal*p* (**A**). The complex branching at the O-6 position of residue **A** was evidenced by NOESY correlations from **D** H1 (δ 4.37), **F** H1 (δ 5.17), and **K** H1 (δ 4.40) to **A** H6 (δ 3.74, 3.82), confirming the respective attachments of →6)-β-D-Gal*p* (**D**), →5)-α-L-Ara*f* (**F**), and →4)-β-D-Xyl*p* (**K**). Furthermore, robust NOESY interactions elucidated the peripheral side-chain architectures: **C** H1 to **D** H6 (δ 4.41/3.75, 3.85) mapped a β-D-Gal*p*-(1→6)-β-D-Gal*p*-(1→ linkage; **I** H1 to **E** H2 (δ 5.22/4.06) connected →2,4)-α-L-Rha*p* (**I**) to →2)-α-L-Rha*p* (**E**); and **J** H1 to **I** H4 (δ 4.57/3.64) verified the terminal β-D-Glc*p* (**J**) decoration on the O-4 position of residue **I**.

**Figure 4:**
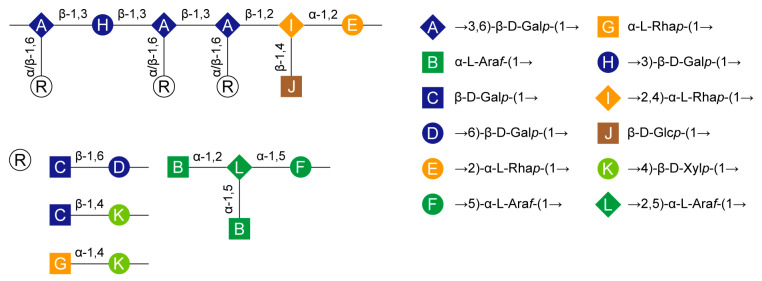
Proposed structural model of the repeating unit of BcP-b2. The topological framework was developed through a thorough integration of GC-MS-based methylation analysis and multi-dimensional NMR spectroscopy (^1^H, ^13^C, COSY, HSQC, HMBC, and NOESY). Due to the large macromolecular weight (~38.1 kDa) and the highly branched, dendritic architecture, a complete representation of the entire polysaccharide chain is not feasible. Instead, this schematic illustrates the primary backbone assembly, principal glycosidic linkage patterns, and determined anomeric configurations (α/β). The symbol ‘R’ designates hyper-branching points or the attachment sites of minor side-chain residues.

By thoroughly integrating the 1D/2D NMR data, methylation profiling, and monosaccharide composition analysis, we established the core structural model of BcP-b2 as a highly branched galactoarabinan. The proposed backbone consists of alternating residues: →3,6)-β-D-Gal*p*-(1→, →2)-α-L-Rha*p*-(1→, →3)-β-D-Gal*p*-(1→, and →2,4)-α-L-Rha*p*-(1→ residues. The complex structure is characterized by heterogeneous side chains, predominantly including β-D-Gal*p*-(1→6)-β-D-Gal*p*-(1→, β-D-Gal*p*-(1→4)-β-D-Xyl*p*-(1→, α-L-Rha*p*-(1→4)-β-D-Xylp-(1→, and an arabinan motif α-L-Ara*f*-(1→2,5)-α-L-Ara*f*-(1→→5)-α-L-Ara*f*-(1→. A discrete β-D-Glc*p*-(1→ branch is attached to the O-4 position of the →2,4)-α-L-Rha*p* scaffold. Due to the large macromolecular weight (~38.1 kDa) and the intensive branching architecture, it is not possible to represent all repeating units in a single schematic. Nonetheless, the identified anomeric configurations and main linkage patterns are summarized in Figure 4, where R indicates points of hyper-branching substitution.

### 3.4. BcP-b2 Mitigates Oxidative Stress In Vitro and In Vivo

**Figure 5:**
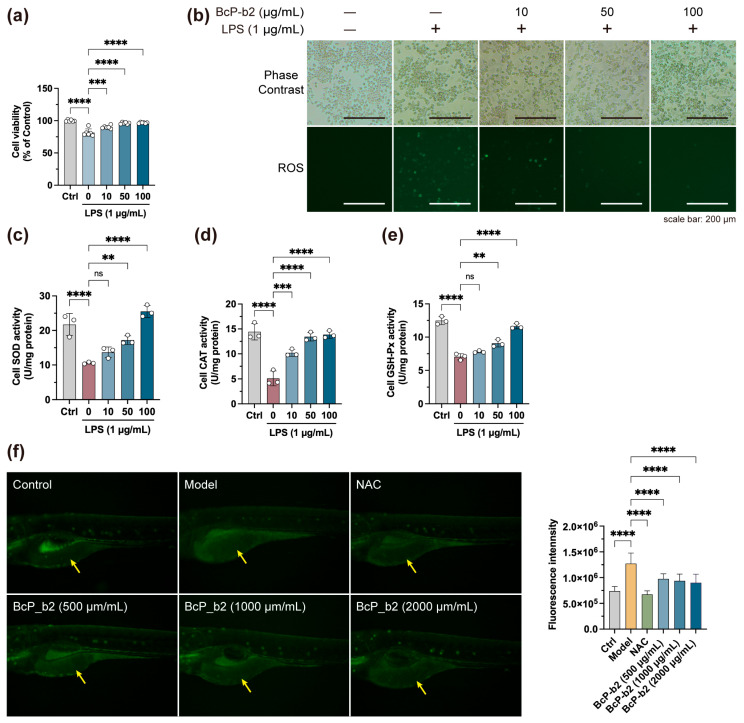
Cytoprotective and antioxidant efficacy of BcP-b2 in vitro and in vivo. (**a**) Protective effect of BcP-b2 against lipopolysaccharide (LPS)-induced cytotoxicity in RAW 264.7 macrophages. Cells were pretreated with BcP-b2 (0, 10, 50, or 100 μg/mL) for 24 h in DMEM with 10% FBS at 37 °C and 5% CO_2_, followed by an LPS challenge (1 μg/mL) for an additional 24 h. Cellular viability was assessed utilizing a Cell Counting Kit-8 (CCK-8) assay (Yeasen, Shanghai, China). Data are presented as the mean ± standard deviation (SD) derived from six technical replicates per condition, pooled from three independent biological experiments. (**b**) Inhibition of LPS-induced intracellular reactive oxygen species (ROS) accumulation in RAW 264.7 macrophages. ROS levels were visualized and quantified using a Reactive Oxygen Species Assay Kit (Beyotime, Shanghai, China) with the DCFH-DA fluorescent probe. Representative fluorescence micrographs are displayed; scale bar = 200 μm. (**c**–**e**) Upregulation of endogenous antioxidant defense enzymes, including superoxide dismutase (SOD), catalase (CAT), and glutathione peroxidase (GSH-Px), in LPS-challenged RAW 264.7 macrophages. Enzyme activities were quantified using their respective commercial assay kits (Beyotime). Data are presented as the mean ± SD derived from three independent biological experiments. For panels (**a**–**e**), statistical significance was evaluated via a one-way analysis of variance (ANOVA) followed by Tukey’s multiple comparisons test using GraphPad Prism 10. ns, no significant difference (*p* > 0.05); **, *p* < 0.01; ***, *p* < 0.001; and ****, *p* < 0.0001. (**f**) Menadione-induced ROS overproduction in a zebrafish embryo model. Albino mutant zebrafish larvae at 3 days post-fertilization (dpf) were pretreated with the positive control *N*-acetylcysteine (NAC; 62.5 μg/mL) or BcP-b2 (500, 1000, or 2000 μg/mL) for 3 h, followed by a 24-h challenge with menadione. Intracellular ROS levels were assessed using CellROX Green Reagent (Invitrogen, Carlsbad, CA, USA). Representative fluorescence images are shown, with arrowheads indicating the yolk sac. Quantification of the relative fluorescence intensity in the yolk sac is presented as the mean ± SD derived from 10 larvae, randomly selected from a cohort of 30. Statistical significance was determined by one-way ANOVA followed by Tukey’s multiple comparisons test. ****, *p* < 0.0001.

Based on preliminary cytotoxicity profiling, BcP-b2 exhibited no harmful effects on the viability of RAW 264.7 macrophages at concentrations up to 1000 μg/mL. Consequently, a low-to-moderate dosage regimen (10, 50, and 100 μg/mL) was established to evaluate its cytoprotective efficacy. After LPS-induced injury, BcP-b2 pretreatment restored cellular viability in a dose-dependent manner (Figure 5a). Mechanistically, this cytoprotection was characterized by a significant reduction in intracellular ROS accumulation (Figure 5b). Furthermore, BcP-b2 significantly increased the catalytic activities of the endogenous antioxidant defense enzymes, specifically SOD, CAT, and GSH-Px (Figure 5c–e). To validate these antioxidant properties in vivo, a zebrafish embryo model was employed. In our pre-experiments, initial maximum tolerated concentration (MTC) screening across a 125–2000 μg/mL gradient identified 2000 μg/mL as the highest non-lethal dose. Therefore, concentrations of 500, 1000, and 2000 μg/mL were selected for subsequent in vivo efficacy evaluations. Consistent with the in vitro findings, BcP-b2 effectively attenuated menadione-induced ROS overproduction, as demonstrated by the dose-dependent reduction in whole-body fluorescence intensity (Figure 5f).

### 3.5. Dual Immunomodulatory Regulation of Inflammatory Mediators

**Figure 6:**
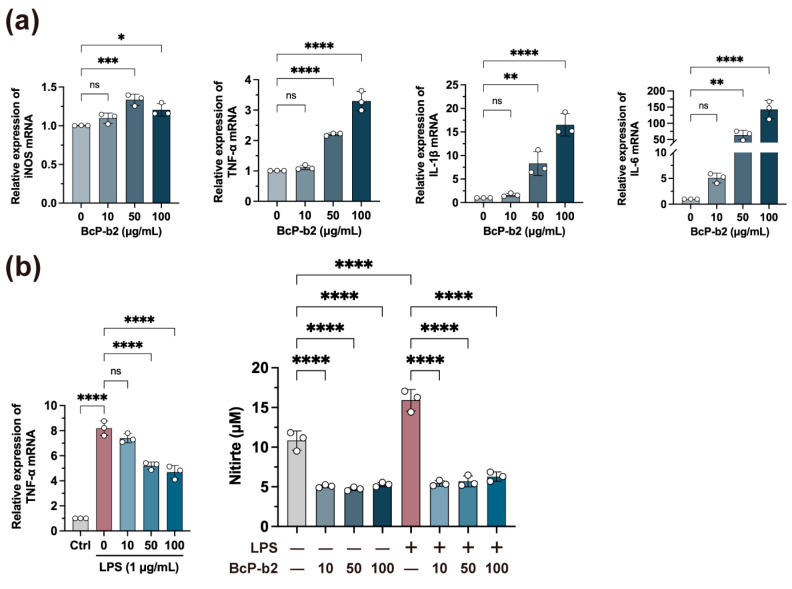
Dual immunomodulatory regulation of inflammatory mediators in RAW 264.7. (**a**) Dose-dependent upregulation of pro-inflammatory marker. The expression of mRNA for pro-inflammatory markers, including iNOS, TNF-α, IL-1β, and IL-6, was assessed in resting macrophages. Cells were incubated with BcP-b2 (0, 10, 50, or 100 μg/mL) for 24 h in DMEM with 10% FBS at 37 °C and 5% CO_2_. Total RNA was isolated utilizing FreeZol Reagent (Vazyme, Nanjing, China), and quantitative real-time PCR (qRT-PCR) was executed using TB Green Premix Ex Taq II (TaKaRa Bio, Beijing, China). (**b**) Inhibition of lipopolysaccharide (LPS)-induced TNF-α transcription and nitric oxide (NO) overproduction. Macrophages were pretreated with the indicated concentrations of BcP-b2 for 24 h, followed by a 24-h challenge with LPS (1 μg/mL). NO production in the culture supernatant was quantified via the Griess reagent assay. For panels (**a**,**b**), data are presented as the mean ± standard deviation (SD) derived from three technical replicates per condition, pooled from three independent biological experiments. Statistical significance was evaluated utilizing a one-way analysis of variance (ANOVA) followed by Tukey’s multiple comparisons test using GraphPad Prism 10. ns, no significant difference (*p* > 0.05); *, *p* < 0.05; **, *p* < 0.01; ***, *p* < 0.001; and ****, *p* < 0.0001.

The immunomodulatory profile of BcP-b2 was assessed by measuring the expression and secretion of key inflammatory mediators in RAW 264.7 macrophages. Under basal physiological conditions, exposure to BcP-b2 at concentrations ranging from 10 to 100 μg/mL resulted in a moderate, dose-dependent upregulation in the mRNA expression of primary pro-inflammatory markers, including iNOS, TNF-α, IL-1β, and IL-6 (Figure 6a). In contrast, in the context of LPS-induced inflammation, BcP-b2 pretreatment significantly attenuated the overproduction of nitric oxide (NO) and strongly suppressed TNF-α transcription (Figure 6b). These varied responses highlight a bidirectional immunomodulatory capacity, wherein BcP-b2 functions as a mild immunostimulant under resting conditions while exhibiting potent anti-inflammatory efficacy during pathological stress.

### 3.6. Transcriptomic Profiling Elucidates the Dual Immunomodulatory Mechanisms of BcP-b2

**Figure 7:**
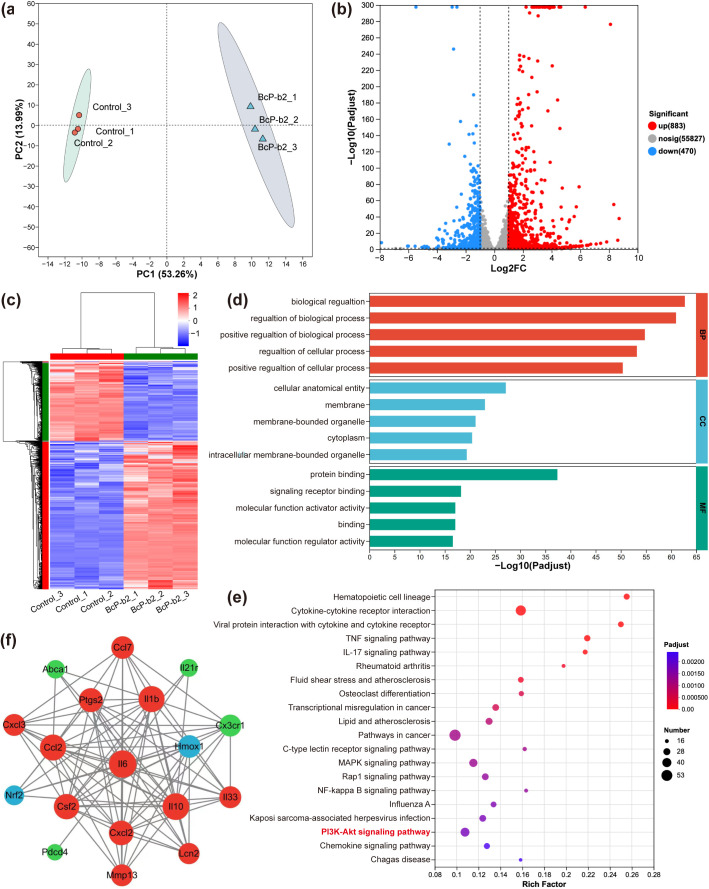
Transcriptomic reprogramming in BcP-b2-treated RAW 264.7 macrophages. Macrophages were incubated with BcP-b2 (50 μg/mL) or a vehicle control (PBS) for 24 h in DMEM with 10% FBS at 37 °C and 5% CO_2_. Independent biological replicates (*n* = 3) were evaluated for each condition. Total RNA was extracted utilizing FreeZol Reagent (Vazyme, Jiangsu, China) and subsequently processed for paired-end sequencing (NovaSeq X Plus, 150 bp, ~50 million reads per sample). Transcriptome libraries were constructed utilizing the Illumina Stranded mRNA Prep Ligation kit (Illumina, San Diego, CA, USA). Differentially expressed genes (DEGs) were determined via DESeq2 (v1.44.0), applying stringent statistical thresholds of a false discovery rate (FDR)-adjusted *p* < 0.05 and an absolute Log_2_ fold change (|Log_2_FC|) > 2. (**a**) Principal component analysis (PCA) of the transcriptomic profiles. PC1 and PC2 denote the first and second principal components, respectively. (**b**) Volcano plot illustrating global differential gene expression. The x- and y-axes represent Log_2_FC and −Log_10_(adjusted *p*-value), respectively. Significantly upregulated (*n* = 883) and downregulated (*n* = 470) transcripts are indicated by red and blue dots, respectively, whereas non-significant transcripts are colored gray. (**c**) Hierarchical clustering heatmap of DEG expression patterns. (**d**) Gene Ontology (GO) functional enrichment analysis. BP, biological process; CC, cellular component; MF, molecular function. (**e**) Kyoto Encyclopedia of Genes and Genomes (KEGG) pathway enrichment analysis, displaying the top 20 significantly enriched pathways. (**f**) Protein–protein interaction (PPI) network of DEGs functionally associated with oxidative stress and inflammation. The network was constructed utilizing the STRING database (v12.0, medium confidence score > 0.4) and visualized via Cytoscape (v3.9.1). Hub genes were prioritized employing the Maximal Clique Centrality (MCC) algorithm within the cytoHubba plugin. Node color definitions: red, upregulated inflammatory mediators; blue, oxidative stress regulators; green, downregulated signaling components.

To investigate the molecular mechanisms of BcP-b2-mediated immunomodulation, RNA sequencing (RNA-seq) was performed on RAW 264.7 macrophages treated with BcP-b2 at a concentration of 50 μg/mL for 24 h. Principal component analysis (PCA) showed a clear distinction in the transcriptomic profiles between the BcP-b2-treated and control groups, indicating significant transcriptional reprogramming (Figure 7a). By applying stringent criteria (adjusted *p* < 0.05 and |Log_2_FC| > 2), we identified 1,353 differentially expressed genes (DEGs), which included 883 upregulated and 470 downregulated transcripts (Figure 7b,c; Table S3). Gene Ontology (GO) enrichment analysis revealed that these DEGs were predominantly enriched in biological processes governing immune responses, inflammatory signaling, and oxidative stress regulation (Figure 7d; Table S4). Additionally, Kyoto Encyclopedia of Genes and Genomes (KEGG) pathway analysis highlighted significant enrichment in the Toll-like receptor (TLR) and PI3K/Akt signaling pathways (Figure 7e; Table S5).

To better understand the transcriptomic changes, the expression profiles of core DEGs implicated in macrophage activation and redox homeostasis were closely examined. Under basal conditions, BcP-b2 significantly upregulated the transcription of IL-1β (Log_2_FC = 5.572, adjusted *p* < 0.0001), CCL2 (Log_2_FC = 6.343, adjusted *p* < 0.0001), and PTGS2 (Log_2_FC = 4.518, adjusted *p* < 0.0001) (Table S3). This transcriptional signature strongly suggests that BcP-b2 primes resting macrophages for an immunocompetent state, which is consistent with the observed elevation in mRNA levels of important pro-inflammatory mediators. This basal priming is a characteristic of polysaccharide-derived immunomodulators, which potentiate innate immune surveillance without triggering excessive inflammatory cytotoxicity.

**Figure 8:**
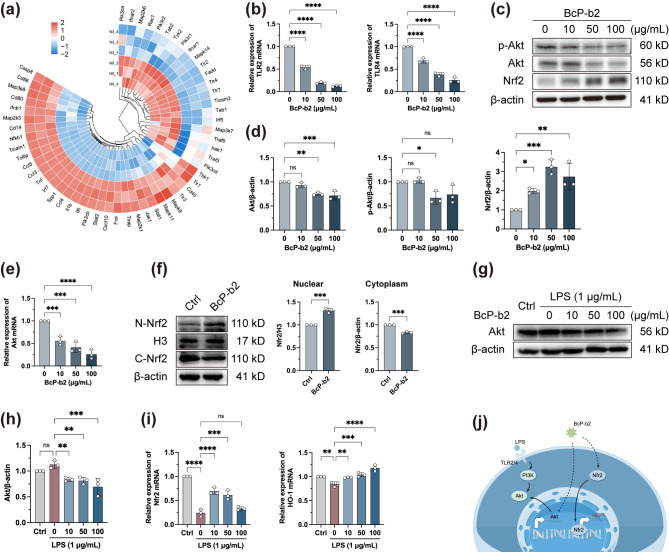
Modulatory effects of BcP-b2 on antioxidant and anti-inflammatory signaling cascades in RAW 264.7 macrophages. (**a**) Transcriptomic profile of the Toll-like receptor (TLR) signaling pathway derived from RNA-seq analysis (*n* = 3 independent biological replicates per group). (**b**) Downregulation of TLR2 and TLR4 mRNA expression following a 24-h incubation with BcP-b2 (0–100 μg/mL). Cells were treated with BcP-b2 at concentrations of 0, 10, 50, or 100 μg/mL for 24 h in DMEM with 10% FBS at 37 °C and 5% CO_2_. Total RNA was extracted using FreeZol Reagent (Vazyme, Jiangsu, China), and qRT-PCR was performed using TB Green Premix Ex Taq II (TaKaRa, Beijing, China). Data are presented as the mean ± standard deviation (SD) derived from three technical replicates per condition, pooled from three independent biological experiments. (**c**,**d**) Representative Western blots and quantification indicate that BcP-b2 inhibited total Akt and phosphorylated Akt (p-Akt) protein levels while promoting Nrf2 expression. Total proteins were extracted using RIPA lysis buffer (Beyotime, Shanghai, China) supplemented with protease and phosphatase inhibitors. The antibodies for p-Akt, Akt, and Nrf2 were obtained from Proteintech (Wuhan, China). GAPDH was used as a loading control. Data are presented as mean ± SD derived from three independent biological replicates. (**e**) BcP-b2 reduced Akt mRNA levels under the same treatment conditions as in (**b**). Data are presented as the mean ± SD derived from three technical replicates per condition, pooled from three independent biological experiments. For panels (**b**–**e**), statistical significance was determined by one-way ANOVA followed by Tukey’s multiple comparisons test using Prism 10 (GraphPad Software, San Diego, CA, USA). ns, *p* > 0.05; *, *p* < 0.05; **, *p* < 0.01; ***, *p* < 0.001; and ****, *p* < 0.0001. (**f**) Nuclear and cytoplasmic Nrf2 protein levels. Cells were treated with 50 μg/mL BcP-b2 for 24 h, and nuclear and cytoplasmic proteins were extracted using a Nuclear and Cytoplasmic Protein Extraction Kit (Beyotime, Shanghai, China). GAPDH and Lamin B1 were used as loading controls for cytoplasmic and nuclear fractions, respectively. Data are presented as mean ± SD derived from three independent biological replicates. Statistical significance was determined by unpaired Student’s *t*-test. ***, *p* < 0.001. N-Nrf2, nuclear Nrf2; C-Nrf2, cytoplasmic Nrf2. (**g**,**h**) Representative Western blots and quantification indicate that BcP-b2 suppressed Akt protein expression in LPS-challenged cells. Cells were pretreated with BcP-b2 at 0, 10, 50, or 100 μg/mL for 24 h and then challenged with LPS (1 μg/mL) for 24 h in DMEM with 10% FBS at 37 °C and 5% CO_2_. GAPDH was used as a loading control. Data are presented as mean ± SD derived from three independent biological replicates. (**i**) BcP-b2 effectively restored Nrf2 and HO-1 mRNA levels in LPS-induced macrophages under the same treatment conditions as in (**g**,**h**). Data are presented as the mean ± SD derived from three technical replicates per condition, pooled from three independent biological experiments. For panels (**g**–**i**), statistical significance was determined by one-way ANOVA followed by Tukey’s multiple comparisons test. ns, *p* > 0.05; **, *p* < 0.01; ***, *p* < 0.001; and ****, *p* < 0.0001. (**j**) Schematic diagram depicting the antioxidant and anti-inflammatory effects of BcP-b2 through PI3K/Akt inhibition and Nrf2/HO-1 activation. Created with Figdraw (www.figdraw.com).

The transcript abundance of Nrf2 remained unaltered under basal conditions, implying that BcP-b2 does not activate the antioxidant defense system in resting cells. However, in response to LPS challenge, BcP-b2 significantly elevated total Nrf2 protein levels and promoted its nuclear translocation (Figure 8c,f). Additionally, it effectively restored the LPS-suppressed expression of Nrf2 and HO-1 mRNAs (Figure 8i). These phase-dependent responses confirm that BcP-b2 functions as a bidirectional modulator. It enhances innate immunity under physiological homeostasis while simultaneously activating the Nrf2/HO-1 antioxidant axis under pathological inflammatory stress. Since Nrf2-driven HO-1 induction is known to reduce ROS accumulation and attenuate NF-κB-mediated transcription, this transcriptomic reprogramming establishes a definitive molecular basis for the dual regulatory capacity of BcP-b2.

To understand the functional topology of these DEGs, a protein–protein interaction (PPI) network was constructed using the STRING database and visualized via Cytoscape (Figure 7f). The network consists of 18 representative nodes and 90 edges, organized into three highly interconnected modules: (i) an inflammatory cytokine–chemokine hub predominantly featuring IL-1β, IL6, CCL2, CCL7, CXCL2, CXCL3, CSF2, MMP13, LCN2, IL10, IL33, and PTGS2; (ii) an oxidative-stress defense node, represented by Nrf2 and HO-1; and (iii) a signal-transduction regulatory cluster, which includes downregulated CX3CR1, ABCA1, IL21R, and PDCD4. Additionally, a topological analysis using the Maximal Clique Centrality (MCC) algorithm identified IL6, IL10, CCL2, IL-1β and CSF2 as the core hub genes. This integration of inflammatory mediators and antioxidant effectors in the network suggests that BcP-b2 provides cytoprotection through a coordinated interplay between inflammatory suppression and antioxidant enhancement.

### 3.7. BcP-b2 Concurrently Modulates the PI3K/Akt and Nrf2/HO-1 Signaling Cascades

Transcriptomic profiling revealed a significant increase in differentially expressed genes (DEGs) regulated by BcP-b2 within the Toll-like receptor (TLR) signaling pathway (Figure 8a). Subsequent quantitative real-time PCR (qRT-PCR) analysis confirmed this increase, demonstrating that BcP-b2 significantly downregulated the transcriptional expression of TLR2 and TLR4 (Figure 8b). Under normal physiological conditions, BcP-b2 reduced total Akt protein abundance and inhibited its active phosphorylation (p-Akt) (Figure 8c,d), which corresponded with a decrease in Akt transcript levels (Figure 8e). Additionally, BcP-b2 upregulated the protein expression of Nrf2 (Figure 8c) and promoted its nuclear translocation (Figure 8f). In the context of LPS-induced inflammatory stress, BcP-b2 significantly suppressed both the transcriptional and translational expression of Akt (Figure 8g,h). Furthermore, it effectively rescued the LPS-mediated suppression of Nrf2 and HO-1 mRNA levels (Figure 8i). Collectively, these correlative findings strongly indicate that the dual cytoprotective and immunomodulatory efficacies of BcP-b2 are mediated via the targeted reduction of the PI3K/Akt inflammatory axis coupled with the robust activation of the Nrf2/HO-1 antioxidant cascade.

## 4. Discussion

This study outlines the structural characteristics of BcP-b2, a homogeneous 38.1-kDa galactoarabinan isolated from *B. cusia*, and investigates its context-dependent immunomodulatory effects through the PI3K/Akt and Nrf2/HO-1 signaling pathways. Unlike previously studied fractions (BCP-1 and BCP-2) [14], which lacked high-resolution sequence and branching data, BcP-b2 is notable for its substantially higher molecular weight and a complex monosaccharide composition that includes various neutral sugars and uronic acids. Multi-dimensional structural analysis revealed that BcP-b2 is a highly branched macromolecule with a defined backbone consisting of →3,6)-β-D-Gal*p*-(1→, →2)-α-L-Rha*p*-(1→, →3)-β-D-Gal*p*-(1→, and →2,4)-α-L-Rha*p*-(1→ residues, along with diverse terminal side chains. This architectural complexity likely influences its bioactivity, as polysaccharides within the 10–1000 kDa range, which possess intricate glycosidic linkages, typically exhibit enhanced receptor-binding affinities [31,32,33]. Moreover, we expanded on previous phenotypic observations by providing targeted signaling validation, demonstrating that BcP-b2 alleviates oxidative and inflammatory stress in both LPS-challenged macrophages and a menaquinone-induced zebrafish model.

BcP-b2 exhibits a unique and context-dependent regulatory phenotype that sets it apart from other well-characterized plant polysaccharides. For instance, while the arabinogalactan from *Larix kaempferi* [32] and *Pollen Typhae* [34] primarily promotes NF-κB-mediated pro-inflammatory signaling, BcP-b2 behaves differently. It primes resting macrophages by upregulating *IL-1β*, *CCL2*, and *PTGS2*, while simultaneously suppressing LPS-induced NO overproduction and Akt phosphorylation. This functional dichotomy is likely due to BcP-b2’s distinctive glycosidic structure. Unlike traditional type II arabinogalactans, BcP-b2 features a rhamnose–xylose branching configuration along with a 1,3,6-linked galactan core. This structure may provide multiple binding sites for innate pattern recognition receptors (PRRs) [35], thus facilitating both immunomodulatory and antioxidant responses. This hypothesis is consistent with established structure–activity relationship (SAR) models, which suggest that factors such as molecular weight, monosaccharide composition, and spatial conformation significantly influence signaling outcomes [15].

The ethnomedicinal classification of *B. cusia* as a “heat-clearing and detoxifying” agent is supported by its capacity to modulate oxidative and inflammatory processes. Similarly to the biological properties reported for other botanical polysaccharides [36,37], BcP-b2 attenuated menaquinone-induced oxidative damage in vivo. These findings indicate that BcP-b2 primarily enhances the body’s own antioxidant defense systems rather than functioning solely as a direct free-radical scavenger.

At the cellular level, BcP-b2 exhibited a dual immunomodulatory profile in macrophages. Under basal physiological conditions, BcP-b2 upregulated the transcription of *iNOS*, *TNF-α*, *IL-1β*, and *IL-6*, thereby priming the cells for an immunocompetent state. This basal activation is a well-known characteristic of polysaccharide-derived immunomodulators and is similar to the priming effects observed with *Brassica rapa* [38] and *Hericium erinaceus* [39]. In contrast, under LPS-induced inflammatory stress, BcP-b2 significantly attenuated NO overproduction and suppressed the transcription of pro-inflammatory mediators. This strong suppression mirrors the established anti-inflammatory mechanisms of the fungal immunomodulatory protein FIP-glu [22], as well as the cytoprotective properties of *Suaeda maritima* polysaccharides [40]. Similarly, macromolecules derived from *Inonotus obliquus* have been found to activate resting M0 macrophages while simultaneously preventing the excessive activation of LPS-polarized M1 macrophages [41]. This functional dichotomy supports a state-dependent immunomodulatory framework, where a single macromolecule can exert different regulatory effects based on the initial activation state of the target cell. In resting macrophages, BcP-b2 enhanced immune surveillance without causing significant cytotoxicity; however, upon LPS challenge, it effectively interrupted the inflammatory amplification. This capacity for context-dependent regulation distinguishes BcP-b2 from conventional immunosuppressants, positioning it as a sophisticated modulator of macrophage plasticity [42].

Transcriptomic profiling provides a molecular framework for understanding functional divergence. The significant baseline induction of IL-1β, CCL2, and PTGS2 prepares resting macrophages for a swift response to pathogens [43] without inappropriately activating the Nrf2/HO-1 axis. Following a subsequent LPS challenge, BcP-b2 downregulated TLR2/4 transcription, while also diminishing PI3K/Akt signaling. This effectively interrupts the inflammatory amplification loop [44,45,46]. At the same time, BcP-b2 promotes Nrf2 nuclear translocation, which helps restore HO-1 expression and enhances antioxidant capacity [47,48,49]. While this targeted pathway modulation strongly correlates with the observed cytoprotection, it is important to note that these findings are associative. Definitive causal validation will require future studies involving genetic knockdown or pharmacological intervention [50].

The structure–activity relationship of BcP-b2 remains associative rather than causal. At 38.1 kDa, BcP-b2 is markedly larger than the previously characterized *B. cusia* fractions BCP-1 (11.6 kDa) and BCP-2 (26.7 kDa), and its primary structure is distinguished by a near-equimolar galactose-to-arabinose ratio (33.12% vs. 29.17%) together with substantial rhamnose and xylose substitution. Polysaccharides in the 10–1000 kDa range commonly exhibit enhanced receptor-binding affinities because chain length supports multivalent interactions with macrophage pattern recognition receptors, including TLR4 [51]. The 1,3,6-linked β-D-galactopyranose core of BcP-b2 serves as a densely branched scaffold, with terminal α-L-arabinofuranose, β-D-galactopyranose, β-D-xylopyranose, and α-L-rhamnopyranose residues projecting from the backbone. This high degree of branching aligns with the architecture of immunostimulatory type II arabinogalactans, in which extensive side-chain substitution, rather than a linear backbone, has been linked to TLR4 engagement and downstream macrophage activation [52,53]. Unlike the classical *Larix* type II motif (1,3-β-D-galactan backbone with 1,6-β-D-galactan side chains), BcP-b2 presents a rhamnose–xylose–arabinose branching pattern that may generate distinct topographical epitopes for innate immune recognition [32]. The resulting high density of terminal residues likely provides multiple low-affinity binding sites for PRRs, enabling BcP-b2 to prime resting macrophages under basal conditions while concurrently suppressing PI3K/Akt signaling and activating the Nrf2/HO-1 axis during LPS-induced inflammatory stress. However, in the absence of enzymatic truncation or site-specific mutagenesis data, the causal contribution of individual glycosidic linkages, particularly the 1,2-linked rhamnose and 1,4-linked xylose branches, to these divergent signaling outcomes cannot be conclusively assigned. Targeted degradation and glycoengineering approaches are therefore required to map the minimal structural epitopes responsible for the dual immunomodulatory and antioxidant activity of BcP-b2.

Several limitations should be acknowledged. First, the three-dimensional structure and complete sequence of all side-chain residues remain incompletely resolved. Second, the mechanistic conclusions drawn lack direct validation through intervention studies. Third, the translational relevance of the findings is limited because they rely on a non-mammalian zebrafish model and an in vitro macrophage system; it is essential to conduct subsequent in vivo validation in mammals. Fourth, the transcriptomic architecture was only mapped under basal conditions, which hinders a complete understanding of the transcriptional reprogramming induced by LPS. Finally, important pharmacokinetic parameters including oral bioavailability, gastrointestinal stability, and systemic immunogenicity, must be systematically evaluated before considering BcP-b2 as a viable therapeutic option [54,55].

Several limitations should be acknowledged. First, the three-dimensional conformation and complete sequence of all side-chain residues remain incompletely resolved, precluding a full understanding of the spatial epitopes recognized by pattern recognition receptors. Second, the mechanistic conclusions are associative rather than causal; definitive validation will require genetic ablation or pharmacological inhibitor studies. Third, the translational relevance of the current findings is constrained by their reliance on a non-mammalian zebrafish embryo model and an in vitro macrophage system. Species differences in physiology, immune architecture, and drug metabolism between teleosts/rodents and humans introduce substantial heterogeneity that limits the predictive value of these preclinical models for clinical efficacy and safety [56]. Validation in mammalian disease models is therefore essential. Fourth, the transcriptomic profiling was performed only under basal conditions, which hinders a complete understanding of the transcriptional reprogramming induced by LPS challenge. Fifth, the inherent structural heterogeneity of plant polysaccharides, manifested as batch-to-batch variation in molecular weight, branching pattern, and monosaccharide composition, poses substantial obstacles for quality control and standardization, representing a major barrier to clinical translation [57]. Finally, the pharmacokinetic behavior of BcP-b2 remains largely uncharacterized. As with most macromolecular polysaccharides, BcP-b2 likely faces poor oral bioavailability, limited intestinal permeability, and rapid gastrointestinal degradation, alongside an undefined in vivo metabolic fate and tissue distribution [55,57]. Systematic evaluation of these parameters, together with comprehensive preclinical safety profiling, is mandatory before BcP-b2 can be advanced toward therapeutic development.

## 5. Conclusions

In conclusion, this study details the targeted isolation and structural analysis of BcP-b2, a homogeneous 38.1-kDa galactoarabinan derived from the roots of *B. cusia* (Nan-Ban-Lan-Gen). Through multi-dimensional analytical techniques, BcP-b2 was identified as a highly branched heteropolysaccharide characterized by a complex backbone composed of →3,6)-β-D-Gal*p*-(1→, →2)-α-L-Rha*p*-(1→, →3)-β-D-Gal*p*-(1→, and →2,4)-α-L-Rha*p*-(1→ residues. Additionally, terminal side chains consist of β-D-Gal*p*, β-D-Xyl*p*, α-L-Rha*p*, and α-L-Ara*f* units. This intricate molecular structure aligns with the essential fine-structural requirements that influence the bioactivity of immunomodulatory polysaccharides [15,31]. Phenotypic evaluations were conducted in vitro using RAW 264.7 macrophages and in vivo using a zebrafish model. Results demonstrate that BcP-b2 effectively reduces intracellular ROS accumulation, enhances the capacities of endogenous antioxidant enzymes such as SOD, CAT, and GSH-Px, and mitigates LPS-induced inflammatory mediator secretion. Mechanistically, integrated transcriptomic profiling and targeted validation suggest that these protective effects are governed by the simultaneous reduction of the PI3K/Akt inflammatory cascade and the activation of the Nrf2/HO-1 antioxidant axis, evidenced by decreased Akt phosphorylation and increased Nrf2 nuclear translocation. These findings provide a strong molecular basis for the ethno-pharmacological application of Nan-Ban-Lan-Gen as a heat-clearing and detoxifying herb, while also expanding the known macromolecular diversity of *B. cusia*. However, it is important to note that our current understanding relies on correlative observations. To establish definitive causality, future studies should employ targeted pharmacological inhibitors or genetic ablation techniques. Furthermore, advancing BcP-b2 toward therapeutic development will require rigorous validation in mammalian disease models, thorough pharmacokinetic profiling, and comprehensive preclinical safety assessments.

## Figures and Tables

**Table 1:** Linkage types of BcP-b2.

RT (min)	Linkages	PMAAs	Mass Fragments (*m*/*z*)	Molar Ratio (%)
10.842	t-Ara(*f*)	1,4-di-*O*-acetyl-2,3,5-tri-*O*-methyl arabinitol	71, 87, 102, 118, 129, 145, 161	10.40
11.382	t-Rha(*p*)	1,5-di-*O*-acetyl-6-deoxy-2,3,4-tri-*O*-methyl mannitol	59, 72, 89, 102, 115, 118, 131, 145, 162, 175	5.41
11.950	t-Ara(*p*)	1,5-di-*O*-acetyl-2,3,4-tri-*O*-methyl arabinitol	88, 101, 102, 117, 118, 129, 161	1.20
12.025	t-Xyl(*p*)	1,5-di-*O*-acetyl-2,3,4-tri-*O*-methyl xylitol	88, 101, 102, 118, 119, 161, 162	1.63
13.798	3-Ara(*f*)	1,3,4-tri-*O*-acetyl-2,5-di-*O*-methyl arabinitol	87, 99, 113, 118, 129, 201, 233	1.22
13.853	t-Man(*p*)	1,5-di-*O*-acetyl-2,3,4,6-tetra-*O*-methyl mannitol	87, 102, 118, 129, 145, 161, 162, 205	1.96
13.914	t-Glc(*p*)	1,5-di-*O*-acetyl-2,3,4,6-tetra-*O*-methyl glucitol	87, 102, 118, 129, 145, 161, 162, 205	4.64
14.133	3-Rha(*p*)	1,3,5-tri-*O*-acetyl-6-deoxy-2,4-di-*O*-methyl mannitol	87, 89, 101, 118, 131, 234	1.32
14.393	2-Rha(*p*)	1,2,5-tri-*O*-acetyl-6-deoxy-3,4-di-*O*-methyl mannitol	89, 100, 115, 130, 131, 175, 190	5.94
14.448	t-Gal(*p*)	1,5-di-*O*-acetyl-2,3,4,6-tetra-*O*-methyl galactitol	87, 102, 118, 129, 145, 161, 162, 205	9.23
14.824	5-Ara(*f*)	1,4,5-tri-*O*-acetyl-2,3-di-*O*-methyl arabinitol	87, 102, 118, 129, 162, 189	5.56
15.119	4-Xyl(*p*)	1,4,5-tri-*O*-acetyl-2,3-di-*O*-methyl xylitol	87, 102, 118, 129, 162, 189	2.92
16.248	3-Man(*p*)	1,3,5-tri-*O*-acetyl-2,4,6-tri-*O*-methyl mannitol	87, 101, 118, 129, 161, 202, 234	4.11
16.405	3-Gal(*p*)	1,3,5-tri-*O*-acetyl-2,4,6-tri-*O*-methyl galactitol	87, 101, 118, 129, 161, 202, 234	4.91
16.638	2-Glc(*p*)	1,2,5-tri-*O*-acetyl-3,4,6-tri-*O*-methyl glucitol	88, 101, 129, 130, 161, 190, 205	1.94
16.939	2,4-Rha(*p*)	1,2,4,5-tetra-*O*-acetyl-6-deoxy-3-*O*-methyl mannitol	88, 101, 117, 130, 143, 190, 203	4.69
17.096	4-Gal(*p*)	1,4,5-tri-*O*-acetyl-2,3,6-tri-*O*-methyl galactitol	87, 102, 113, 118, 129, 162, 233	2.04
17.260	4-Glc(*p*)	1,4,5-tri-*O*-acetyl-2,3,6-tri-*O*-methyl glucitol	87, 102, 113, 118, 129, 162, 233	2.33
17.404	2,5-Ara(*f*)	1,2,4,5-tetra-*O*-acetyl-3-*O*-methyl arabinitol	87, 88, 129, 130, 189, 190	2.44
17.486	6-Glc(*p*)	1,5,6-tri-*O*-acetyl-2,3,4-tri-*O*-methyl glucitol	87, 99, 102, 118, 129, 162, 189, 233	1.86
17.603	2,4-Xyl(*p*)	1,2,4,5-tetra-*O*-acetyl-3-*O*-methyl xylitol	87, 88, 129, 130, 145, 146, 189, 190	1.16
18.547	6-Gal(*p*)	1,5,6-tri-*O*-acetyl-2,3,4-tri-*O*-methyl galactitol	87, 99, 102, 118, 129, 162, 189, 233	7.22
21.045	3,6-Glc(*p*)	1,3,5,6-tetra-*O*-acetyl-2,4-di-*O*-methyl glucitol	87, 101, 118, 129, 189, 202, 234	1.20
21.606	3,6-Gal(*p*)	1,3,5,6-tetra-*O*-acetyl-2,4-di-*O*-methyl galactitol	87, 101, 118, 129, 160, 189, 234	14.66

**Table 2:** The ^1^H and ^13^C NMR chemical shifts of the sugar residues of BcP-b2.

Code	Glycosyl Residues	Chemical Shifts (ppm)					
		H1/C1	H2/C2	H3/C3	H4/C4	H5/C5	H6/C6
A	→3,6)-β-D-Gal*p*-(1→	4.42	3.48	3.66	4.07	3.86	3.74, 3.82
		103.38	70.73	80.18	68.41	73.64	66.57
B	α-L-Ara*f*-(1→	5.02	4.06	3.88	4.02	3.66	/
		107.5	80.79	76.54	84.03	59.42	/
C	β-D-Gal*p*-(1→	4.41	3.3	3.71	3.84	3.6	3.68, 3.76
		103.24	73.14	70.1	68.24	72.66	61.31
D	→6)-β-D-Gal*p*-(1→	4.37	3.5	3.72	4.04	3.84	3.75, 3.85
		103.44	73.75	72.1	70.18	73.71	66.86
E	→2)-α-L-Rha*p*-(1→	5.21	4.06	3.94	3.39	3.72	1.18
		98.39	76.31	70.16	75.76	68.64	16.57
F	→5)-α-L-Ara*f*-(1→	5.17	4.15	3.94	4.06	3.88	/
		109.23	81.64	76.68	83.84	65.85	/
G	α-L-Rha*p*-(1→	5.05	3.73	3.83	3.36	3.8	1.23
		101.08	70.1	70.41	71.98	69.96	16.82
H	→3)-β-D-Gal*p*-(1→	4.47	3.59	3.69	3.9	3.62	3.82
		103.14	69.97	80.12	68.8	75.03	60.95
I	→2,4)-α-L-Rha*p*-(1→	5.22	4.08	3.98	3.64	3.94	1.18
		98.7	76.09	70.36	80.39	68.81	16.46
J	β-D-Glc*p*-(1→	4.57	3.45	3.63	4	3.89	3.6
		103.78	71.51	75.11	70.36	73.55	61.06
K	→4)-β-D-Xyl*p*-(1→	4.4	3.22	3.48	3.72	3.3, 4.03	/
		101.64	72.81	73.63	76.25	62.94	/
L	→2,5)-α-L-Ara*f*-(1→	5.14	4.07	4.18	3.82	3.83	/
		106.98	83.93	82.11	81.77	66.21	/

## Data Availability

All data generated or analyzed during this study are included in this published article and/or its Supplementary Materials. The original data presented in the study are openly available in SRA database at accession number PRJNA1469768.

## Supplementary Materials

The following supporting information can be downloaded at https://www.mdpi.com/article/10.3390/antiox15060770/s1. Table S1: Primer Sequences for qRT-PCR; Table S2: The free radicals scavenging activity of BcP-b1 and -b2; Table S3: Upregulated (top 100) and downregulated (top 100) differentially expressed genes; Table S4: List of Gene Ontology (GO) terms; Table S5: List of Kyoto Encyclopedia of Genes and Genomes (KEGG) terms; Supplementary Data: EI-MS fragmentation patterns of partially methylated alditol acetates (PMAAs).

## Abbreviations

The following abbreviations are used in this manuscript:

Ara	arabinose
CAT	catalase
D2O	deuterium oxide
DEGs	differentially expressed genes
DPPH	1,1-diphenyl-2-picrylhydrazyl
FT-IR	Fourier transform infrared
Fuc	fucose
GSH-Px	glutathione peroxidase
Gal	galactose
Gal-UA	galacturonic acid
GC-MS	gas chromatography–mass spectrometry
Glc	glucose
Glc-UA	glucuronic acid
HO-1	heme oxygenase 1
HPAEC	high-performance anion exchange chromatography
ITS	internal transcribed spacer
Man	mannose
Mw	molecular weight
NAC	N-acetyl-cysteine
NJ	neighbor-joining
Nrf2	nuclear factor erythroid 2-related factor 2
•O^2−^	superoxide anion
•OH	hydroxyl radical
p-Akt	phosphorylated Akt
PCA	principal component analysis
PMAA	partially methylated alditol acetate
qRT-PCR	quantitative real-time polymerase chain reaction
Rha	rhamnose
SEM	scanning electron microscope
SOD	superoxide dismutase
TCM	traditional Chinese medicine
TLR	Toll-like receptor
UV	ultraviolet
WB	Western blotting
Xyl	xylose
