# Supplementary material for: Structural Characterization of a Novel Galactoarabinan from Baphicacanthus cusia and Its Protective Effects Against Oxidative Stress and Inflammation via the PI3K/Akt and Nrf2/HO-1 Signaling Axes
Source: Antioxidants (Basel). 2026 Jun 19;15(6):770. doi: 10.3390/antiox15060770 (PMC13295634; doi:10.3390/antiox15060770)
Supplement: Supplementary file 1 [file antioxidants-15-00770-s001.zip › antioxidants-4336095-table S1.pdf]

Table S1. Primer Sequences for qRT-PCR.

	forward primer	reverse primer
iNOS	5'-GGATCCAGTGGTCCAACCTG-3'	5'-GTTGCCATTGTTGGTGGCAT-3'
TNF-α	5'-TTCTATGGCCCAGACCCTCA-3'	5'-ACAAGGTACAACCCATCGGC-3'
IL-1β	5'-GTGTCTTTCCCGTGGACCTT-3'	5'-AATGGGAACGTCACACACCA-3'
IL-6	5'-ACTTCACAAGTCGGAGGCTT-3'	5'-TGCAAGTGCATCATCGTTGT-3'
IL-10	5'-TGAATTCCCTGGGTGAGAAGC-3'	5'-GACACCTTGGTCTTGGAGCTTA-3'
Nrf2	5'-ATGGACTTGGAGTTGCCACC-3'	5'-GCTCATAGTCCTTCTGTCGCT-3'
HO-1	5'-CCTCACAGATGGCGTCACTT-3'	5'-AAGCTGAGAGTGAGGACCCA-3'
GAPDH	5'-ATGGGTGTGAACCACGAGAA-3'	5'-CAGTGATGGCATGGACTGTG-3'
