# Supplementary material for: Structural Characterization of a Novel Galactoarabinan from Baphicacanthus cusia and Its Protective Effects Against Oxidative Stress and Inflammation via the PI3K/Akt and Nrf2/HO-1 Signaling Axes
Source: Antioxidants (Basel). 2026 Jun 19;15(6):770. doi: 10.3390/antiox15060770 (PMC13295634; doi:10.3390/antiox15060770)
Supplement: Supplementary file 1 [file antioxidants-15-00770-s001.zip › antioxidants-4336095-table S2.pdf]

Table S2. The free radicals scavenging activity of BcP-b1 and -b2

Concentration (mg/mL)	Radical scavenging activities (%)					
	DPPH		• OH		• O_2^-	
	BcP-b1	BcP-b2	BcP-b1	BcP-b2	BcP-b1	BcP-b2
1	2.749	4.318	/	/	/	52.482
10	2.293	6.729	1.391	6.528	31.326	60.090
